# Characterizing reference genes for high-fidelity gene expression analysis under different abiotic stresses and elicitor treatments in fenugreek leaves

**DOI:** 10.1186/s13007-024-01167-6

**Published:** 2024-03-16

**Authors:** Amin Ebrahimi, Shahrokh Gharanjik, Elham Azadvari, Sajad Rashidi-Monfared

**Affiliations:** 1 Agronomy and Plant Breeding Department, Faculty of Agriculture, Shahrood University of Technology, Semnan, Iran; 2 Department of Plant Breeding and Biotechnology, Faculty of Agricultural Engineering, Shahrood University of Technology, Shahrood, Iran; 3 Horticultural Sciences Department, Faculty of Agriculture, Shahrood University of Technology, Shahrood, Iran; 4 Plant Breeding and Biotechnology Department, Faculty of Agriculture, Tarbiat Modares University, Tehran, Iran

**Keywords:** Housekeeping gene, Real-time PCR, Fenugreek, Abiotic stress, Elicitors

## Abstract

**Background:**

Quantifying gene expression is a critical aspect of applied genomics research across all organisms, and real-time PCR has emerged as a powerful tool for this purpose. However, selecting appropriate internal control genes for data normalization presents specific challenges. This study aimed to identify suitable reference genes for gene expression analysis under various conditions, encompassing salinity, low and high-temperature stresses, and different elicitor treatments. These treatments included titanium dioxide, cold plasma, 24-epibrassinolide, and melatonin, resulting in a total of 13 unique treatments and 148 treatment combinations applied to fenugreek plants.

**Results:**

As per the analysis performed with the BestKeeper tool, *EEF-1α*, and *GAPDH* were recognized as the most stable reference genes under the majority of conditions. Furthermore, the GeNorm and NormFinder tools identified *β-tubulin* and *EEF-1α* as the most stable reference genes. The findings of this research demonstrated that, although the stability of three reference genes expression was acceptable in almost all evaluated treatments, fluctuations in their expression were observed under the treatments of cold stress with TiO_2_ NPs application, cold plasma application with salinity stress, and cold plasma application with high-temperature stress compared to others. Simultaneously, the GeNorm analysis results demonstrated that in the mentioned treatments, relying on only one reference gene is inadequate. To corroborate the results, we examined the expression profile of the *SSR* gene, a pivotal gene in diosgenin biosynthesis, under all investigated treatments and treatment combinations. The outcomes suggested that employing stable reference genes yielded highly consistent results.

**Conclusions:**

The varying expression patterns of the target genes emphasize the crucial need for precise optimization of experimental conditions and selecting stable reference genes to achieve accurate results in gene expression studies utilizing real-time PCR. These findings offer valuable insights into the selection of appropriate reference genes for gene expression analysis under diverse conditions using real-time PCR.

**Supplementary Information:**

The online version contains supplementary material available at 10.1186/s13007-024-01167-6.

## Introduction

The biotic and abiotic stresses significantly impact global crop productivity and food security. Addressing the complexities of biotic and abiotic stresses is essential for maintaining stable crop production systems and safeguarding food security worldwide. A critical aspect in gaining insights into resistance mechanisms and advancing the development of stress-tolerant cultivars lies in the examination of plant responses to stress conditions [1, 2]. The pivotal role of alterations in gene expression in adapting plants to varying environmental conditions and their resilience to stresses underscores the significance of genomic studies and gene expression analysis. These efforts are of utmost importance in advancing genetic engineering for the development of stress-tolerant cultivars. Through the examination of plant behavior, specifically changes in the expression of target genes, and evaluating their performance under abiotic stresses, genetic engineering has played a pivotal role in enhancing plant resistance against adverse conditions [2]. Employing various elicitors, including titanium dioxide nanoparticles (TiO_2_ NPs), 24-epibrassinolide (EBR), cold plasma, and melatonin in plants, has been recognized as a highly effective strategy for alleviating the impacts of environmental stress on plants [3]. In-depth investigations have established the affirmative influence of these elicitors on diverse regulatory and cellular processes within plants. These encompass cell division, longitudinal growth, replication, transcription, translation, membrane and cell wall stability, chromatin organization, ribosome formation, and programmed cell death [4–9].

The assessment and analysis of gene expression play a crucial role in the field of biological research. Quantitative polymerase chain reaction (qPCR) serves as a practical and valuable tool for validating gene data. When compared to alternative evaluation methods like Northern blot hybridization and reverse transcription polymerase chain reaction (RT-PCR), qPCR exhibits notable advantages, including enhanced sensitivity, specificity, and a wide quantitative range spanning up to seven orders of magnitude. The accuracy of gene expression studies relies heavily on the stability of reference gene expression across different experimental conditions. To ensure precise results, it is essential to appropriately normalize the data obtained from qPCR reactions conducted in diverse experimental conditions and tissues. Despite the availability of various strategies for normalizing qPCR data, achieving proper normalization remains one of the primary challenges associated with this method. The process of identifying suitable reference genes for qPCR analysis has garnered significant attention, despite being time-consuming and requiring substantial effort [10]. Typically, the selection of an ideal reference gene involves identifying candidate genes and subsequently evaluating their expression stability under the desired experimental conditions. However, alternative methods employed for reference gene selection may possess certain limitations and drawbacks. An illustrative example of such limitations is the unavailability of microarray datasets for all species [11]. Recent studies indicate that no single reference gene universally exhibits consistently high expression stability across all biological investigations.

Fenugreek (*Trigonella foenum-graceum* L.) is an annual herbaceous plant belonging to the Leguminosae family, originally hailing from the eastern Mediterranean region. Renowned for its production of medicinal alkaloids, steroid compounds, sapogenins, and notable therapeutic properties. Extensive research has been conducted to explore the therapeutic effects and chemical compounds associated with fenugreek, contributing to our understanding of its medicinal potential [12]. Extensive research has demonstrated that abiotic stresses exert a profound impact on the morphological, physiological, biochemical, and molecular characteristics of fenugreek [4, 6, 13]. These changes result from the varied responses of fenugreek genes to these stresses. Beyond abiotic stresses, the effects of significant and practical elicitors, both individually and in combination with non-biotic stresses, have been investigated to examine their influence on the morphological, physiological, biochemical, and molecular traits of fenugreek [4, 6, 13]. These investigations offer valuable insights into the intricate dynamics between fenugreek and its surrounding environment, illuminating the plant’s adaptive responses to diverse stressors. The findings from these studies indicate that the application of these elicitors, particularly at optimal concentrations, has resulted in enhanced tolerance of fenugreek. This improvement can be attributed to modifications in the content of primary and secondary metabolites in fenugreek, influenced by alterations in gene expression. The outcomes of numerous studies have demonstrated that subjecting fenugreek to abiotic stresses and the mentioned elicitors, at optimal concentration, and during the appropriate growth stage, have effectively elevated the content of diosgenin [4, 6, 7, 13]. Diosgenin stands out as one of the most crucial and valuable metabolites in fenugreek.

To comprehend the stress adaptation mechanisms and conduct genetic investigations on this plant, it is imperative to identify dependable and consistent reference genes under various experimental conditions. Until now, comprehensive studies on the stability of candidate reference genes for fenugreek have not been undertaken under the conditions employed. Hence, the goal of this study is to identify the most dependable internal control genes from a set of candidate genes (*EEF-1α, UBE2D2, β-tubulin, Actin 11, HSP70, GAPDH, UbcH10, eIF4A, 25 S rRNA*, and *18 S rRNA*) to normalize the qPCR results in fenugreek under diverse stress conditions (such as salinity, low and high-temperature stresses) and various stimulating treatments (including titanium dioxide (TiO_2_ NPs), cold plasma, 24-epibrassinolide (EBR), and melatonin). Furthermore, this study assessed the expression profile of the sterol side chain reductase (*SSR*) gene, which plays a crucial role in the biosynthesis of diosgenin, by performing normalization data using both the most stable and unstable candidate reference genes under all experimental conditions considered.

## Materials and methods

### Plant materials

The fenugreek seeds (Boshruyeh genotype) underwent sterilization through immersion in a 2% (v/v) sodium hypochlorite solution for 10 min, followed by three rinses with distilled water.

### Cold plasma treatment

In this study, the fenugreek seeds were subjected to treatment using a dielectric barrier discharge (DBD) system. Plasma was generated by utilizing argon as the working gas, with a frequency of 13 kHz and a pulse duration of 2 µs between two electrodes. For each treatment, three biological replicates were conducted, with 100 healthy seeds selected for each replicate. Half of the seeds were exposed to the plasma jet at two different voltages (2000 and 4000 V) and various exposure times (0, 1, 2, and 4 min), while the remaining seeds were left untreated [7]. Following the treatment, the fenugreek seeds were promptly planted in pots measuring 35 * 25 * 20 cm, filled with a consistent blend of peat moss, perlite, and sand. These pots were situated in a growth chamber under controlled conditions, featuring a 16/8 h (day/night) photoperiod, a temperature range of 22–25 ^º^C, a relative humidity set at 60–65%, and a light intensity of 400 µmol m^-2^.

### EBR treatment

At 4 weeks post-planting, the fenugreek seedlings, at the 6-leaf stage, were divided into four groups. These groups were subsequently sprayed with varying concentrations of EBR (0, 4, 8, and 16 µM), with a repeat application after 6 h. The EBR stock solution (Sigma-Aldrich, USA), with a final concentration of 100 µM, was prepared by dissolving 4.8 mg of EBR in 3 mL of ethanol within a 200 mL volumetric flask. The final volume was adjusted to 100 mL using double-distilled water, resulting in a final concentration of 100 µM. The lower EBR concentrations (4, 8, and 16 µM) were prepared by diluting the stock solution, and 0.1% surfactant (Tween 20) was added before the spraying. Each pot received an equal amount of EBR spray (10 mL), ensuring comprehensive coverage of the plant surface [6]. Solvent solutions matching the concentrations of the reagent dilutions were employed as controls in all experiments. Each treatment was assigned three biological replicates. The EBR utilized in this study was acquired from Sigma-Merck Company (CAS number: 78821-43-9).

### TiO_2_ NPs treatment

TiO_2_ NPs with a predominance of the anatase form (99.7% purity) and an average size of 20–30 nm were obtained from Sigma-Aldrich, USA. Solutions containing these TiO_2_ NPs were prepared at concentrations of 0, 2, 5, and 10 ppm. The preparation process involved using filtered, double-distilled water, based on preliminary experiments. To facilitate the dissolution of TiO_2_ and create a nanoscale solution, the mixture underwent ultrasonic treatment (Elmasonic P 60 H model, Avantor company, Germany) for 45 min, while keeping the mixture in a dark environment. Seedlings at the six-leaf stage underwent spraying with three concentrations of TiO_2_ NPs solution (2, 5, and 10 ppm) along with 0.1% surfactant (Tween 20), with a repeated application after 6 h. Each pot received an equivalent volume of TiO_2_ NPs spray (10 mL), ensuring comprehensive coverage of the plant surface. To maintain consistent conditions, the control group was sprayed with double-distilled water. Each treatment was assigned three biological replicates.

### Melatonin treatment

To prepare the melatonin solutions, the solute was initially dissolved in ethanol, and subsequently, the compound was diluted with distilled water [ethanol/water (v/v) = 1/10,000]. Upon reaching the six-leaf stage, different doses of melatonin (0, 30, 60, and 90 ppm) were introduced into the irrigation water and applied to the pots for 7 days. These concentrations were selected based on preliminary experiments. The control group, receiving no melatonin, underwent irrigation with a mixture of ethanol and distilled water in a ratio of 1 in 10,000 [4]. Each pot received an equal amount of the melatonin solution (100 ml). The melatonin employed in this study was obtained from Sigma-Merck Company (EC number: 200–797-7, CAS number: 73–31 − 4). Each treatment was assigned three biological replicates.

### Low and high temperature treatments

After undergoing TiO_2_, EBR, and melatonin treatments, the plants, including both plasma-treated seeds and untreated seeds, were categorized into four groups based on their exposure to stress. The first group served as the control and was kept under normal conditions in a growth chamber (23 °C). The second and third groups were exposed to cold and high-temperature stresses, and placed in a growth chamber at 10 and 42 °C, respectively, for two durations (6 and 24 h) [6]. In broad terms, the research treatments in this section encompass (comprising both plasma-treated and untreated seeds):

- Varied voltages (2000 and 4000 V), diverse exposure times (0, 1, 2, and 4 min), different TiO_2_ concentrations (0, 2, 5, and 10 ppm), and distinct temperatures (23 °C, 10 °C, and 42 °C for 6 and 24 h).
- Different voltages (2000 and 4000 V), varying exposure times (0, 1, 2, and 4 min), multiple concentrations of EBR (0, 4, 8, and 16 µM), and diverse temperatures (23 °C, 10 °C, and 42 °C for 6 and 24 h).
- Varied voltages (2000 and 4000 V), diverse exposure times (0, 1, 2, and 4 min), various melatonin levels (0, 30, 60, and 90 ppm), and different temperatures (23 °C, 10 °C, and 42 °C for 6 and 24 h). According to the treatments applied, this study was conducted as a factorial in a completely randomized design with three replications. Subsequently, the leaves from each treatment were collected, and preserved at -80 °C.

### Salinity treatment

Following the application of TiO_2_, EBR, and melatonin, the fourth group, comprising both plasma-treated and untreated seeds, underwent exposure to a salinity stress of 200 mM for one week. This daily amount of 100 ml for each pot was determined according to the previous study of our research team and preliminary experiments [4, 13]. The applied treatments in this section encompass (comprising both plasma-treated and untreated seeds):

- Varied voltages (2000 and 4000 V), diverse exposure times (0, 1, 2, and 4 min), different concentrations of TiO_2_ (0, 2, 5, and 10 ppm), along with salinity stress (200 mM).
- Different voltages (2000 and 4000 V), varying exposure times (0, 1, 2, and 4 min), multiple concentrations of EBR (0, 4, 8, and 16 µM), in addition to salinity stress (200 mM).
- Different voltages (2000 and 4000 V), diverse exposure times (0, 1, 2, and 4 min), various melatonin levels (0, 30, 60, and 90 ppm), combined with salinity stress (200 mM). According to the treatments applied, this study was conducted as a factorial in a completely randomized design with three replications. Subsequently, the leaves from each treatment were collected, and preserved at -80 °C.

### RNA extraction and cDNA synthesis

RNA extraction was performed using the RNeasy® Plant Mini Kit (QIAGEN, Venlo, The Netherlands) in accordance with the manufacturer’s guidelines. To remove DNA contamination, one microgram of RNA extract was treated with DNase I (Thermo Fisher, Waltham, Massachusetts). The quality and quantity of the extracted RNA were assessed using a nanodrop spectrophotometer (ND-2000 ultra-micro nucleic acid protein analyzer- Thermo, Waltham, MA, USA) and 1% agarose gel electrophoresis. To generate the first-strand cDNA, the PrimeScript^TM^RT master mix (Code No. RR036A, Takara, Kyoto, Japan) was used in conjunction with 1 µg of total RNA. The RNA samples extracted from leaf tissue, as assessed on a 1% agarose gel and compared to a standard weight, demonstrated suitable quantity and quality for cDNA synthesis (Supplementary Fig. 1).

### Reference genes selection

Based on previous studies [5, 8, 9, 14, 15], ten different reference genes were selected for this research and their accuracy was checked using Oligo Analyzer v.3.1 (http://eu.idtdna.com/calc/analyzer). As per earlier research findings, these primers were chosen from genes associated with the family Leguminosae or the Trigonella genus. The names and sequences of these primers and alongside the *SSR* gene as the target gene, are detailed in Table 1. Primer specificity was confirmed through the melting curve analysis of the RT-qPCR reaction.

### Real-time polymerase chain reaction

Real-time PCR was performed using SYBR® Green PCR Master Mix 2X (Ampliqon, Danish manufacturer) and the real-time PCR system (Applied Biosystems Q7, Waltham, MA, USA). Each reaction consisted of a 10 µL volume with three biological and two technical replicates. To prevent contamination, a negative control (including all reaction components except for cDNA) was included at every stage of the experiment. The qRT-PCR program included 35 cycles of 95 °C for 20 s and 61 °C for 40 s preceded by 95 °C for 10 min. The threshold cycle (Cq) was automatically measured, and correlation coefficients (R^2^) along with slope were computed using a standard curve generated from a tenfold series dilution of the cDNA templates. Subsequently, the respective qRT-PCR efficiencies (E) for each gene were calculated based on the provided slope. The melt curve analysis of the ten reference genes revealed a consistent single peak across different treatments. The amplification curves exhibited robust repeatability, indicating that the primers successfully amplified a singular PCR product. As a result, these reference genes are deemed suitable for detailed qPCR analyses (Supplementary Fig. 2).

### Evaluation of target gene expression using stable and unstable reference genes

To validate the findings obtained from assessing the expression stability of reference genes, the expression profile of the *SSR* gene, a key gene involved in diosgenin biosynthesis, was analyzed. This section utilized the *EEF-1α*, *β-tubulin*, *EEF-1α* + *β-tubulin*, and *GAPDH* genes as the most stable genes, while the *UbcH10* and *25 S rRNA* genes employed as the least stable genes, serving as internal controls across all experimental treatments. RNA extraction was performed using the RNeasy® Plant Mini Kit (QIAGEN, Venlo, The Netherlands) in accordance with the manufacturer’s guidelines. To remove DNA contamination, one microgram of RNA extract was treated with DNase I (Thermo Fisher, Waltham, Massachusetts). Real-time PCR was performed using SYBR® Green PCR Master Mix 2X (Ampliqon, Danish manufacturer) and the real-time PCR system (Applied Biosystems Q7, Waltham, MA, USA). Each reaction consisted of a 10 µL volume with three biological and two technical replicates. The qRT-PCR program included 35 cycles of 95 °C for 20 s and 61 °C for 45 s preceded by 95 °C for 15 min. Before analyzing the expression data of the target gene, thorough examinations of melting and amplification curves were conducted to ensure the accuracy of the data. Furthermore, the real-time PCR data were evaluated utilizing the relative standard curve method, relying on the 2^^ΔΔCt^ equation.

### Data analysis

The PCR efficiency was determined by using the Cq values obtained from three replicates of each sample and the standard curve derived from serial dilutions. Additionally, the melting curve was served as a specific proliferation factor for the products. GeNorm [16], BestKeeper [17], and NormFinder [18] tools were employed to ascertain the most suitable reference gene and identify the most stable gene expression values. In this study, we employed BestKeeper tool to estimate the standard deviation values (SD) and variation coefficient (CV) of each reference gene based on their Cq values. The reference genes with the lowest SD and CV are considered to exhibit more stable expression levels. BestKeeper tool directly employs the Cq value for stability analysis, avoiding an additional conversion step.

In the analysis using GeNorm and NormFinder tools, the Cq values were transformed into relative quantities through the formula 2^−∆Ct^ [19]. GeNorm tool evaluates the stability of reference genes by calculating the stability measurement (M) value, where a lower M value indicates higher stability of the reference gene [17]. The software considers M values below 1.5 as indicative of stable expression. The determination of the optimal number of reference genes was based on the paired coefficient of variation Vn/Vn + 1. Normally, when the Vn/Vn + 1 value falls below 0.15, adding a new reference gene is considered unnecessary [18]. However, if it exceeds this threshold, the inclusion of the (*n* + 1)^th^ reference gene becomes necessary. In this research, the GeNorm algorithm was employed to assess the stability of reference gene expression in fenugreek plants subjected to various stress conditions. Additionally, the study evaluated the stability of candidate reference genes using the NormFinder tool, which ranks them according to their variability in gene expression within and between groups. NormFinder integrates the direct change value or stability value of reference genes to identify the most stable genes, ranking those with the least variability as the best ones [16, 20]. Graphs were generated using Prism 8.0 software. According to the treatments applied, this study was conducted as a factorial in a completely randomized design with three replications. Furthermore, the Duncan test was carried out to compare the means using SPSS 26 software a 1% level (IBM SPSS, Armonk, NY, USA).

**Table 1:** Candidate reference genes and primers sequences used for real-time-qPCR

Gene names	Gene description	Primer 5’-3’	Correlation coefficient (R^2^)	qRT-PCR efficiency (%)	Annealing temperature (C^º^)	Accession number
*eEF-1α*	Eukaryotic elongation factor 1-alpha	TTTCACTCTTGGTGTGAAGCAGAT GACTTCCTTCACGATTTCATCGTAA	0.999	99.9	61–61	gi|351,734,545|
*UBE2D2*	Ubiquitin-conjugating enzyme E2	ATTGCCTGCTGATCCTGATCTGC ACCACTGCAACCACACCAAGC	0.945	92	60–61	MT822516
*β-tubulin*	Beta-tubulin	GCTGACCACACCTAGCTTTGG AGGGAACCTTAGGCAGCATGT	0.986	98.9	60–60	MT822512
*Actin 11*	Actin 11	CAGCCACACTGTCCCCATCTA AGCAAGGTCGAGACGAAGGA	0.989	99.5	60–60	gi|255,684,847|
*HSP70*	Heat shock protein	CTTGGTGCGTCAGCGTATCT GCGGTGCCATTGTGTTTCAT	0.989	96.5	61 − 60	NM_001357341.1
*GAPDH*	Glyceraldehyde-3-phosphate dehydrogenase	AAGCCAGCATCCTATGATCAGATT CGTAACCCAGAATACCCTTGAGTTT	0.994	105.3	61–61	MT822517
*UbcH10*	Ubiquitin 10	TGGTCAGTAATCAGCCAGTTTGG GCACCACAAATACTTGACGAACAG	0.970	103.3	61–61	gi|351,727,992|
*eIF4A*	Eukaryotic initiation factor 4a	CATGGATGTACCTGTGGTGAAAC CTGTCAGCAGAAGGTCCTCATTA	0.999	105.5	60–61	AK073620
*25 S rRNA*	25 S ribosomal RNA	AAGGCCGAAGAGGAGAAAGGT CGTCCCTTAGGATCGGCTTAC	0.980	96	60–60	AK119809
*18 S rRNA*	18 S ribosomal RNA	CTACCACATCCAAGGAAG CAATTACCAGACACTAACG	0.997	98.7	61 − 60	gi|343,347|
*SSR*	Sterol side chain reductase	AGGTGGGAGATATGCTAGAATG CATTCTGTGTGTCTCCCTGCC	-	-	61 − 60	MK060115.1

## Results

PCR efficiency and correlation coefficient data for the considered genes are summarized in Table 1. The results indicated that the PCR efficiency for the reference genes ranged from 92 to 105.5%, with an R^2^ between 0.94 and 0.99. As per the MIQE guidelines, the acceptable range for PCR efficiency falls between 80% and 120%, with an ideal value of 100%. In conclusion, the study’s results indicate that the RNA extracted from leaf tissue samples is appropriate for cDNA synthesis.

### Expression stability analysis of selected reference genes under different conditions

**Fig. 1:**
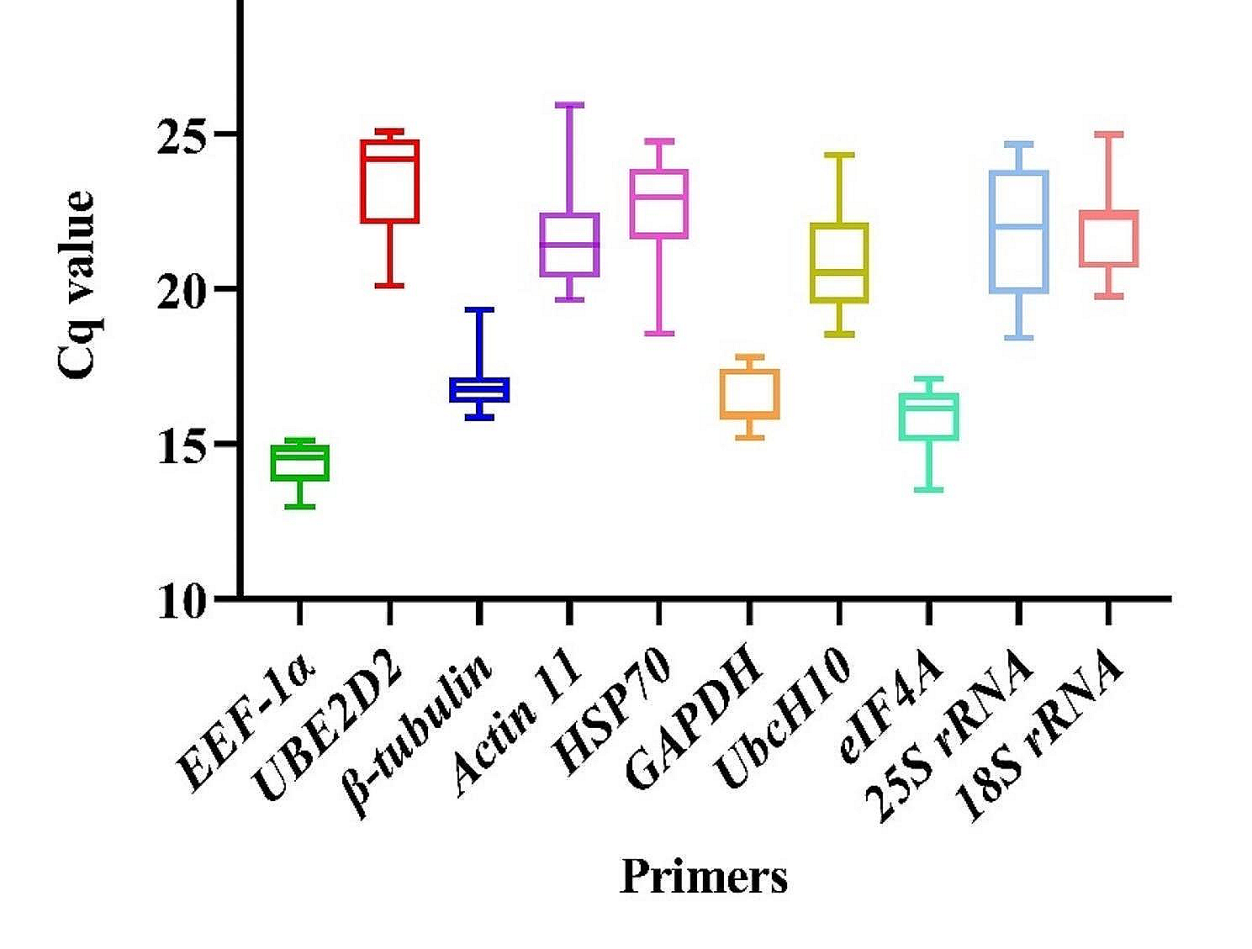
Quantification cycle (Cq) values of ten candidate reference genes under all experimental treatments

Examining 13 treatments and 148 distinct treatment combinations, this study aimed to identify the most stable reference genes among a set of 10 commonly used one. The preliminary assessment of the expression stabilities of the ten candidate reference genes was indicated by the Cq, reflecting the transcript abundance of the genes in the samples under investigation. As depicted in Fig. 1, the mean Cq values of the reference genes ranged from 14.4 to 23.5. Our results showed that the *EEF-1α* gene had the highest expression level across all samples, with a mean Cq of 14.4 ± 0.68 (mean ± SD).

In contrast, the *25 S rRNA* gene had the lowest expression level, with a mean Cq of 21.60 ± 2.86. In addition to employing Cq values for evaluating gene expression stability, we performed comprehensive data analysis using well-established statistical tools including BestKeeper, NormFinder, and GeNorm. This methodology was utilized to confirm our findings and gain a more thorough insight into the dataset. Using these statistical tools, we assessed the stability of reference genes across diverse experimental conditions, providing essential guidance in selecting the most appropriate reference genes for accurate gene expression analyses.

### BestKeeper analysis

The outcomes derived from the BestKeeper tool provide significant observations regarding the stability of reference gene expression under various experimental conditions in fenugreek plants, potentially assisting in the identification of reliable reference genes for subsequent studies (Table 2). Our results revealed that among all experimental samples, *EEF-1α* had the most stable reference gene expression with the lowest CV value (4.80%), followed by *GAPDH* (5.60%). In contrast *25 S rRNA, UbcH10*, *Actin 11* and were the most unstable genes with CV values of 13.20%, 12.50%, 10.70% and respectively (Table 2).

Upon examining treatments individually across various experimental conditions (treatment combinations), we noted nearly identical outcomes. The findings imply uniformity in Cq values across the 13 experimental treatments for the *EEF-1α* gene, excluding the TiO_2_ NPs application + cold stress, and cold plasma application + high-temperature and salinity treatments. This lack of notable distinctions extends to the *β-tubulin* and *GAPDH* genes. However, significant differences were observed for these reference genes in the cold plasma application + high-temperature stress and TiO_2_ NPs application + cold stress treatments compared to the other treatments. Contrarily, there was considerable variability in the Cq values among the 13 experimental treatments for the *25 S rRNA* gene, underscoring noteworthy distinctions. This marked divergence in Cq values concerning the 13 experimental treatments was also noted in the *UbcH10* and *Actin 11* genes (Table 3). In samples under salt stress with melatonin treatments, *EEF-1α* exhibited the most stable expression with a CV value of 0.62%, while *25 S rRNA* was the most unstable gene with a CV value of 2.75%. Similarly, under EBR application with cold stress, *EEF-1α* showed the most stable expression with a CV value of 0.55%, while *18 S rRNA* was the least stable (CV = 2.99%). Likewise, under high-temperature stress with TiO_2_ NPs application, *EEF-1α* was the most stable gene with a CV value of 0.39%. However, *25 S rRNA* exhibited the least stable expression with a CV value of 4.11% (Table 3).

### GeNorm analysis

**Fig. 2:**
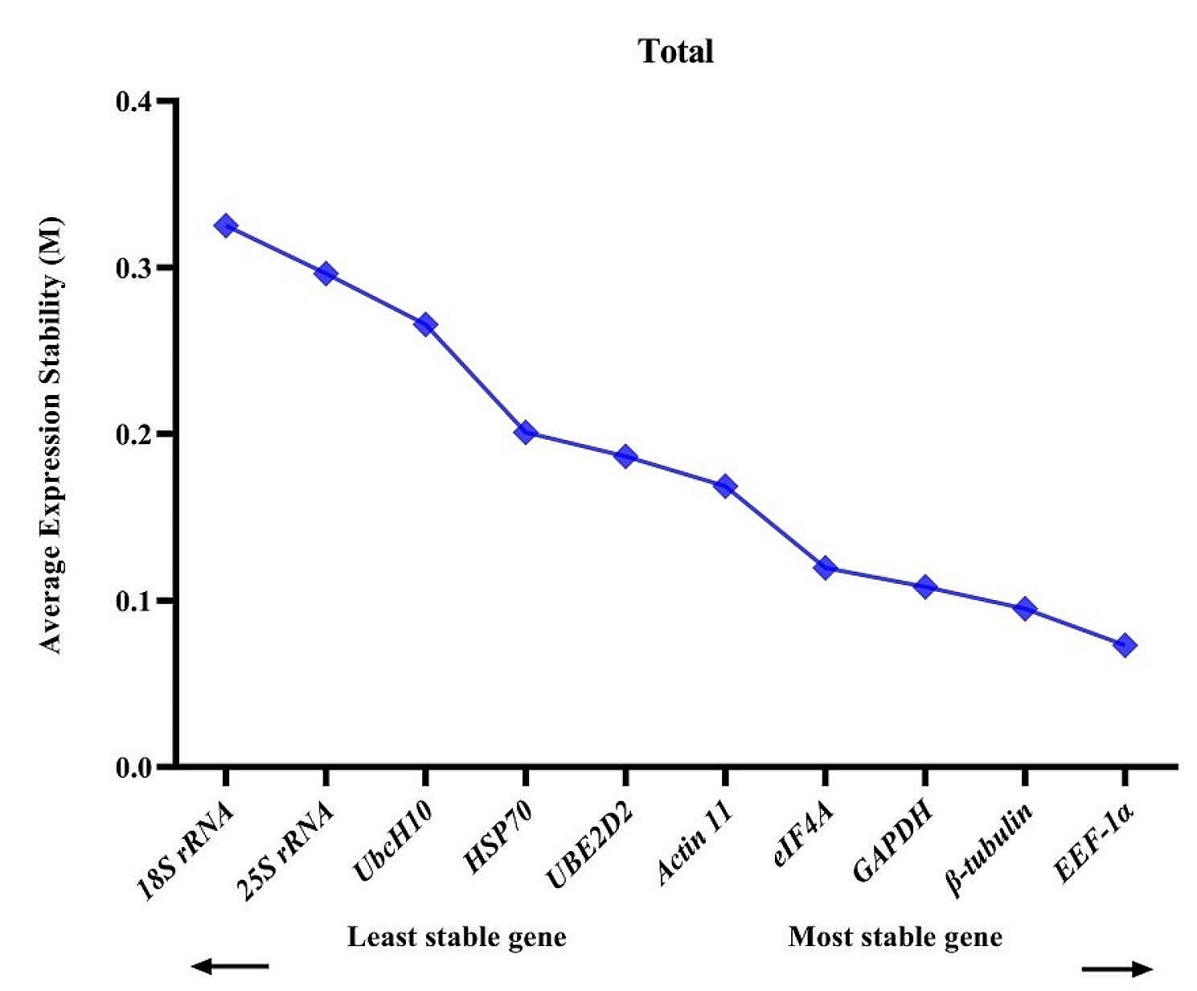
Gene expression stability values (M) and ranking of ten reference genes under all treatments altogether as assayed by GeNorm

The findings indicated that *EEF-1α*, *β-tubulin* and *GAPDH* reference genes were the most stable reference genes with M values of 0.07, 0.09, and 0.11 respectively, when considering treatment combinations together. On the other hand, *UbcH10* (0.32), *25 S rRNA* (0.27), and *18 S rRNA* (0.32) displayed the lowest stability, as evidenced by the highest M values among all treatments. Our findings revealed that all examined genes exhibited an average expression stability value below 1.5, signifying their appropriateness as reference genes for gene expression analysis under stress conditions in fenugreek plants (Fig. 2).

**Fig. 3:**
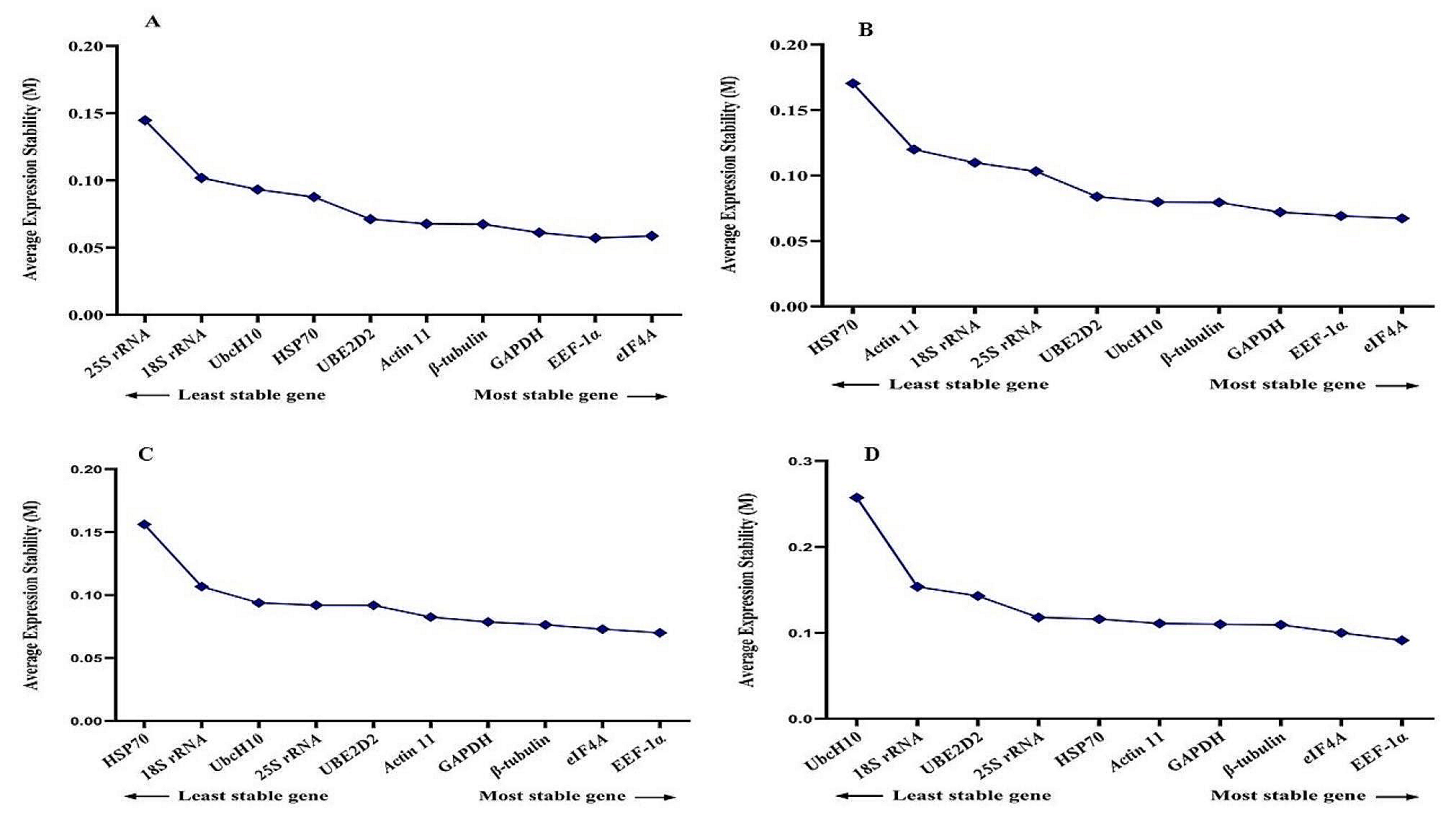
Gene expression stability values (M) and ranking of ten reference genes under salinity stress as assayed by GeNorm. **A** Control conditions, **B** Melatonin + salinity stress, **C** Melatonin + cold stress, **D** Melatonin + high-temperature stress

**Fig. 4:**
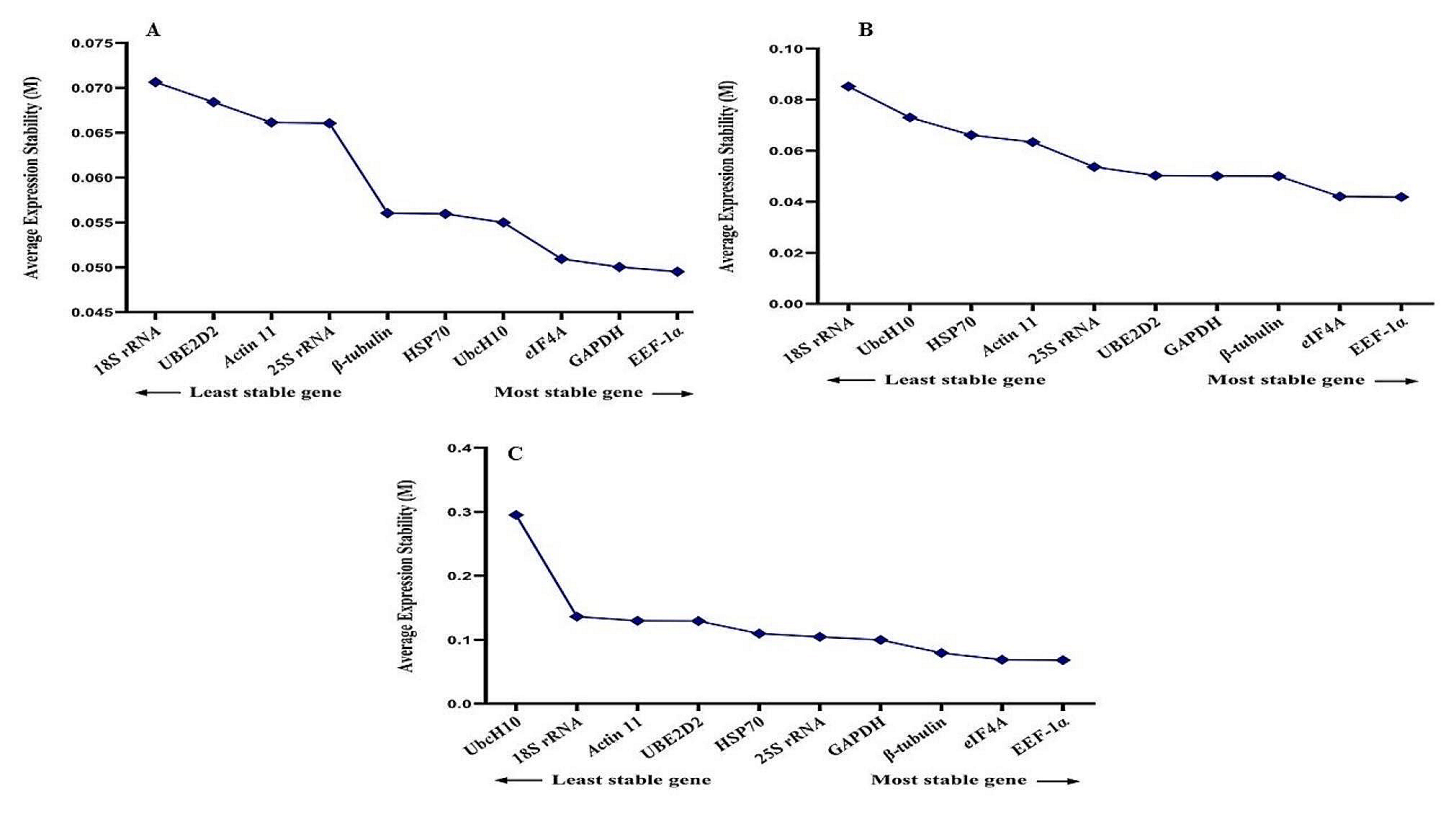
Gene expression stability values (M) and ranking of ten reference genes under cold stress as assayed by GeNorm. **A** EBR + salinity stress, **B** EBR + cold stress, **C** EBR + high-temperature

Taking into account that an ideal reference gene should maintain stable expression across diverse experimental conditions, each of the 13 different treatments was individually analyzed using the GeNorm tool. During melatonin application with salinity stress, *EEF-1α, eIF4A*, and *GAPDH* emerged as the most stable reference genes, while *HSP70, 18 S rRNA*, and *ACT 11* were identified as the least stable genes (Fig. 3). Similarly, under melatonin application with high-temperature and cold stresses, *EEF-1α, eIF4A*, and *β-tubulin* were recognized as the most stable reference genes, whereas *UbcH10, 18 S rRNA*, and *25 S rRNA* were deemed the least stable genes (Fig. 3). Under EBR application with salinity stress, *EEF-1α, eIF4A*, and *GAPDH* were identified as the most stable reference genes, while *25 S rRNA, 18 S rRNA*, and *ACT 11* were deemed the least stable genes (Fig. 4). Similarly, under melatonin application with high-temperature, *EEF-1α, eIF4A*, and *β-tubulin* were recognized as the most stable reference genes, whereas *UbcH10, 18 S rRNA*, and *ACT 11* were considered the least stable genes. Additionally, in the melatonin application with cold stress treatment, *EEF-1α, eIF4A*, and *β-tubulin* were acknowledged as the most stable reference genes, while *UbcH10, 18 S rRNA*, and *HSP70* were identified as the least stable genes (Fig. 4).

**Fig. 5:**
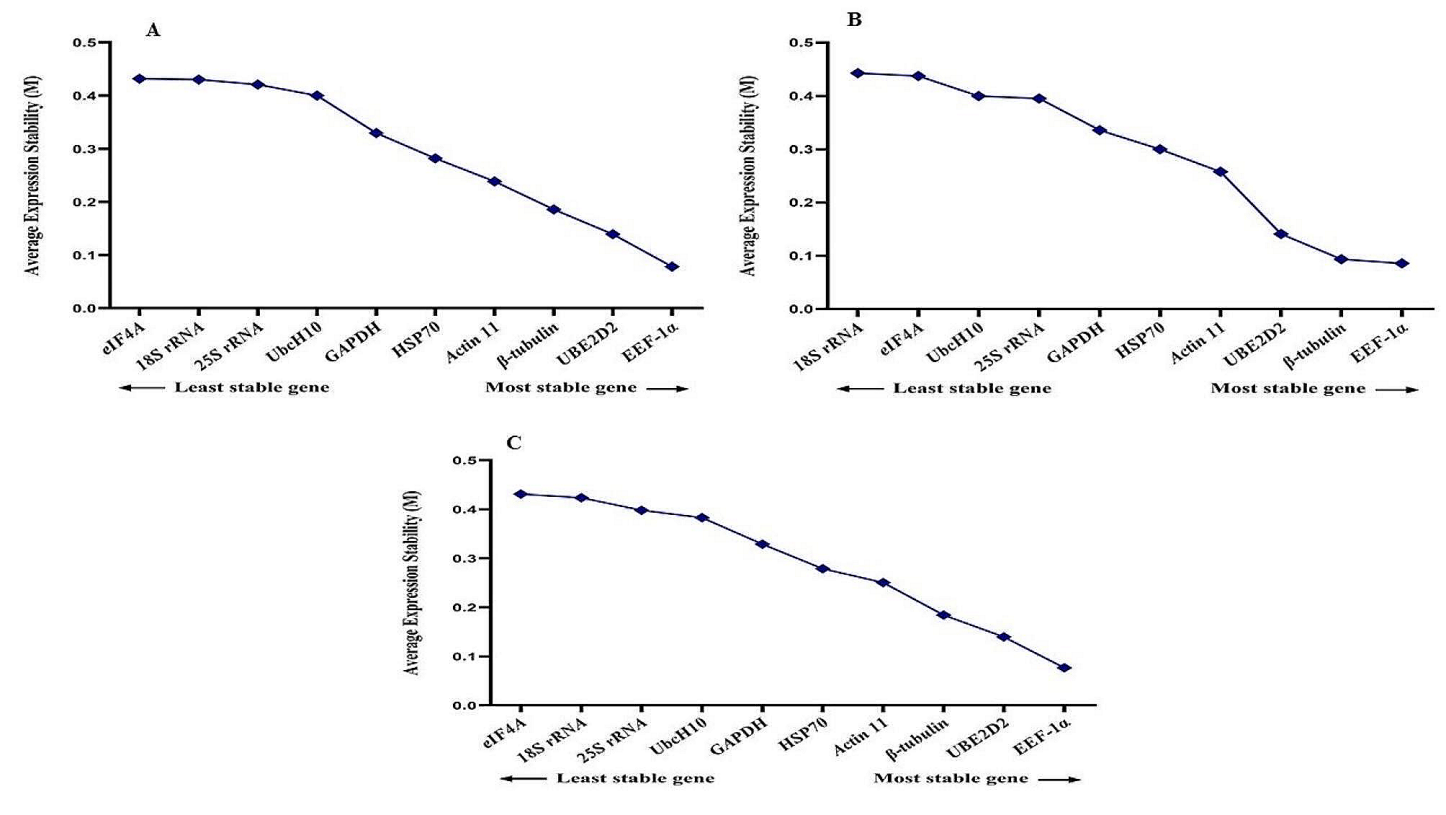
Gene expression stability values (M) and ranking of ten reference genes under high-temperature stress as assayed by GeNorm. **A** TiO_2_ NPs + salinity stress, **B** TiO_2_ NPs + cold stress, **C** TiO_2_ NPs + high-temperature stress

**Fig. 6:**
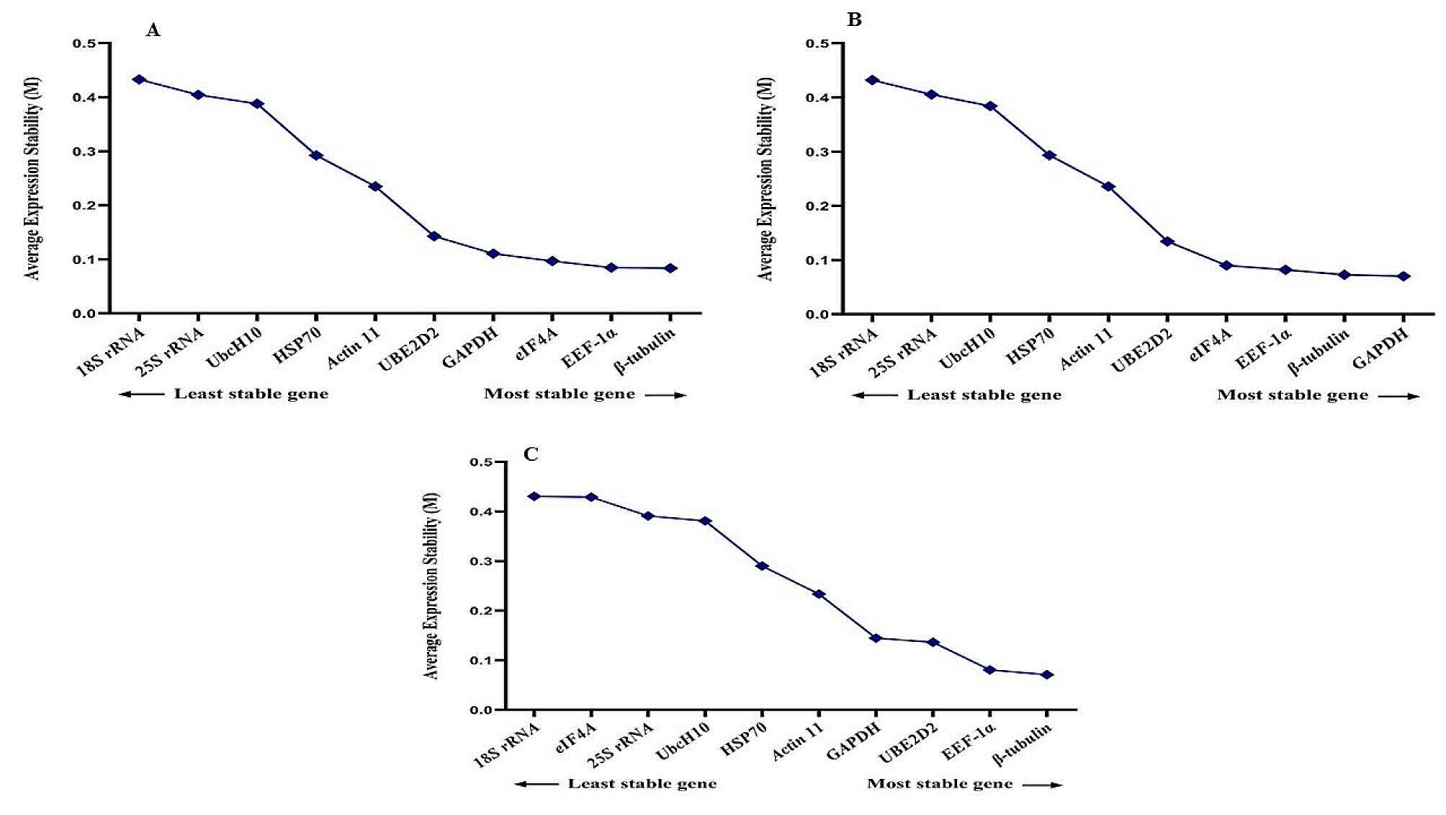
Gene expression stability values (M) and ranking of ten reference genes under high-temperature stress as assayed by GeNorm. **A** Cold plasma + salinity stress, **B** Cold plasma + cold stress, **C** Cold plasma + high-temperature stress

During TiO_2_ application with salinity, high-temperature, and cold stresses, *EEF-1α, β-tubulin*, and *UBE2D2* were identified as the most stable reference genes, while *eIF4A, 18 S rRNA, 25 S rRNA*, and *UbcH10* were noted as the least stable genes (Fig. 5). Similarly, under cold plasma application with salinity, high-temperature, and cold stresses, *EEF-1α, GAPDH*, and β-tubulin were highlighted as the most stable reference genes, while *UbcH10, 18 S rRNA*, and *25 S rRNA* were classified as the least stable genes (Fig. 6).

**Table 2:** Analysis of ten candidate reference genes using the BestKeeper algorithm based on quantification cycle

Treatment	Cq									
	*EEF-1α*	*UBE2D2*	*β-tubulin*	*Actin 11*	*HSP70*	*GAPDH*	*UbcH10*	*eIF4A*	*25 S rRNA*	*18 S rRNA*
Control	14.67 ab	22.30 c	17.50 b	26.00 b	21.00 d	16.00 a	17.00	14.50 d	19.00 f	19.60 e
Melatonin + Cold stress	14.73 ab	24.92 b	17.75 b	21.92 de	22.52 c	16.15 a	22.57 bc	16.32 bc	23.92 bc	23.34 b
Melatonin + High-temperature stress	14.71 ab	24.35 b	17.46 b	23.57 cd	24.63 b	16.00 a	20.21 e	17.78 ab	21.78 de	23.42 b
Melatonin + Salinity stress	14.74 ab	24.91 b	17.77 b	24.18 c	24.44 b	15.74 a	24.32 b	15.98 c	22.28 d	22.06 c
EBR + Cold stress	14.75 ab	24.62 b	17.77 b	21.70 de	23.11 b	15.90 a	27.12 a	16.61 bc	23.67 c	17.10 f
EBR + High-temperature stress	14.89 ab	19.10 e	17.83 b	23.66 cd	23.92 b	16.00 a	20.49 d	16.65 bc	20.35 e	21.71 cd
EBR + Salinity stress	14.95 ab	27.94 a	17.86 b	22.67 d	18.11 e	16.06 a	21.24 cd	14.05 d	19.70 ef	22.51 c
TiO_2_ NPs + Cold stress	13.00 b	23.09 bc	17.37 b	22.32 d	23.78 b	14.00 b	15.97 f	15.98 c	22.24 d	22.40 c
TiO_2_ NPs + High-temperature	14.64 ab	21.9 cd	17.23 b	23.39 cd	23.36 b	15.99 a	21.09 d	18.43 a	15.91 g	23.25 b
TiO_2_ NPs + Salinity stress	14.93 ab	24.26 b	17.59 b	21.45 de	23.52 b	16.00 a	22.65 bc	16.53 bc	24.67 b	23.34 b
Cold plasma + Cold stress	14.85 ab	24.13 b	17.70 b	22.05 d	23.90 b	15.33 a	20.59	15.46 c	20.66 e	22.60 bc
Cold plasma + High-temperature stress	15.55 a	22.81 c	19.74 a	17.22 f	28.66 a	15.98 a	22.24 c	16.74 bc	18.91 f	22.15 c
Cold plasma + Salinity stress	15.48 a	22.91 c	17.43 b	28.51 a	23.02 bc	16.00 a	21.54 cd	15.94 c	27.29 a	27.82 a
Max [Cq]	15.55	27.94	19.74	28.51	28.66	16.00	27.12	18.43	27.29	27.82
Min [Cq]	13.00	19.1	17.46	17.22	18.11	14.00	15.97	14.05	15.91	17.10
Geo Mean [Cq]	14.4	23.5	17.60	22.86	23.38	16.07	21. 54	16.24	21.60	22.46
SD [± Cq]	0.68	2.08	1.10	2.33	2.00	0.90	2.70	1.13	2.86	2.11
CV [%Cq]	4.80	8.75	6.20	10.70	8.40	5.60	12.50	6.75	13.20	9.35
Coeff. of Corr. [r]	0.99	0.89	0.99	0.81	-0.19	0.97	0.90	0.96	0.95	0.95
p-value	0.001	0.001	0.001	0.34	0.05	0.001	0.001	0.001	0.005	0.004

**Table 3:** Analysis of ten candidate reference genes based CV for 13 different experimental treatments

Treatment	CV [%]									
	*EEF-1α*	*UBE2D2*	*β-tubulin*	*Actin 11*	*HSP70*	*GAPDH*	*UbcH10*	*eIF4A*	*25 S rRNA*	*18 S rRNA*
Control	0.80	1.28	1.10	3.14	2.89	1.08	4.80	1.45	4.54	1.46
Melatonin + Cold stress	0.77	1.47	0.53	0.37	1.81	1.07	1.45	1.37	1.88	2.02
Melatonin + High-temperature stress	0.89	0.84	1.04	1.91	2.32	1.22	2.91	0.97	3.01	2.59
Melatonin + Salinity stress	0.62	1.48	1.03	1.86	2.23	0.78	2.43	0.77	2.75	1.48
EBR + Cold stress	0.55	2.16	1.15	1.13	1.57	0.79	2.57	1.76	2.89	2.99
EBR + High-temperature stress	0.21	1.16	1.15	1.04	1.19	0.95	5.97	0.88	2.36	2.63
EBR + Salinity stress	0.89	1.31	0.66	1.08	0.51	1.28	2.47	0.31	1.91	1.46
TiO_2_ NPs + Cold stress	0.37	1.41	1.17	1.73	2.47	1.53	4.86	1.02	1.90	2.53
TiO_2_ NPs + High-temperature	0.39	0.68	1.21	0.88	1.97	1.30	3.89	1.15	4.11	2.26
TiO_2_ NPs + Salinity stress	0.62	0.84	0.91	1.45	1.74	0.94	1.26	0.99	1.87	1.92
Cold plasma + Cold stress	0.76	0.51	0.71	2.41	1.88	0.37	1.51	0.71	2.76	2.16
Cold plasma + High-temperature stress	1.05	0.63	1.81	1.90	2.73	1.87	1.69	1.27	1.97	2.81
Cold plasma + Salinity stress	1.03	1.78	0.20	0.81	2.55	0.77	2.57	0.28	1.87	3.44

**Fig. 7:**
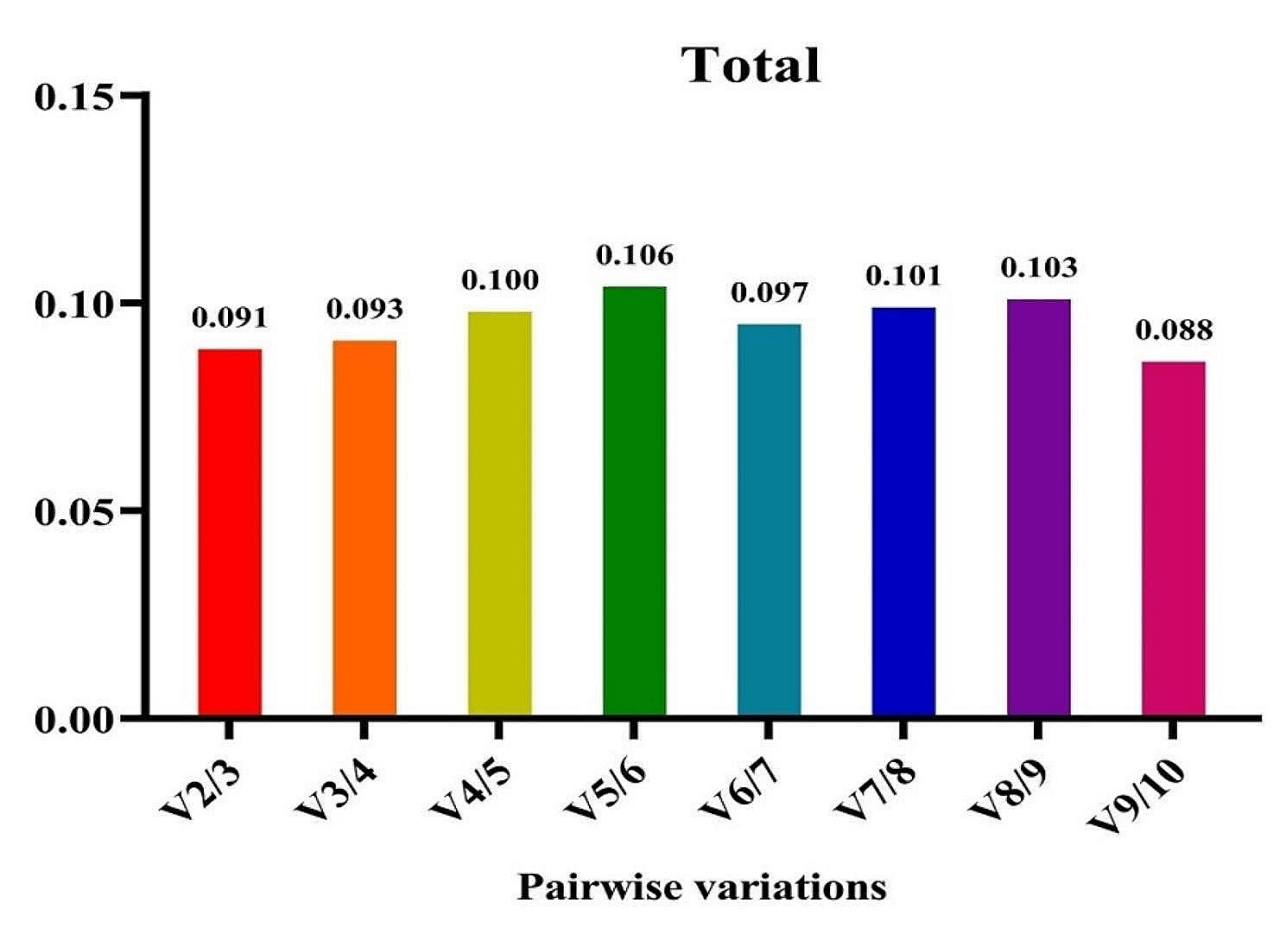
The pairwise variation values of ten reference genes under all treatment together obtained using GeNorm analysis

Recognizing that reference gene expression may differ across various tissues and growth conditions, it is advisable to employ multiple stable reference genes to enhance the precision of gene expression analysis. The GeNorm tool calculates a Normalization Factor (NF) based on the pairwise variation between sequential normalization factors to determine the optimal number of reference genes needed for accurate normalization. Vandesompele et al. (2002) established that the optimal cut-off points for pairwise variation (V) is 0.15, indicating that the inclusion of additional reference genes is unnecessary if the V value is below this threshold [16]. In all experimental treatments, the V2/3 value remained below 0.15, signifying that the utilization of *EEF-1α* and *β-tubulin* genes alone is sufficient and dependable for gene expression analysis (Fig. 7). Thus, our findings suggest that the *EEF-1α* and *β-tubulin* genes are the most appropriate reference genes for accurate gene expression analysis in fenugreek plants across various treatments.

**Fig. 8:**
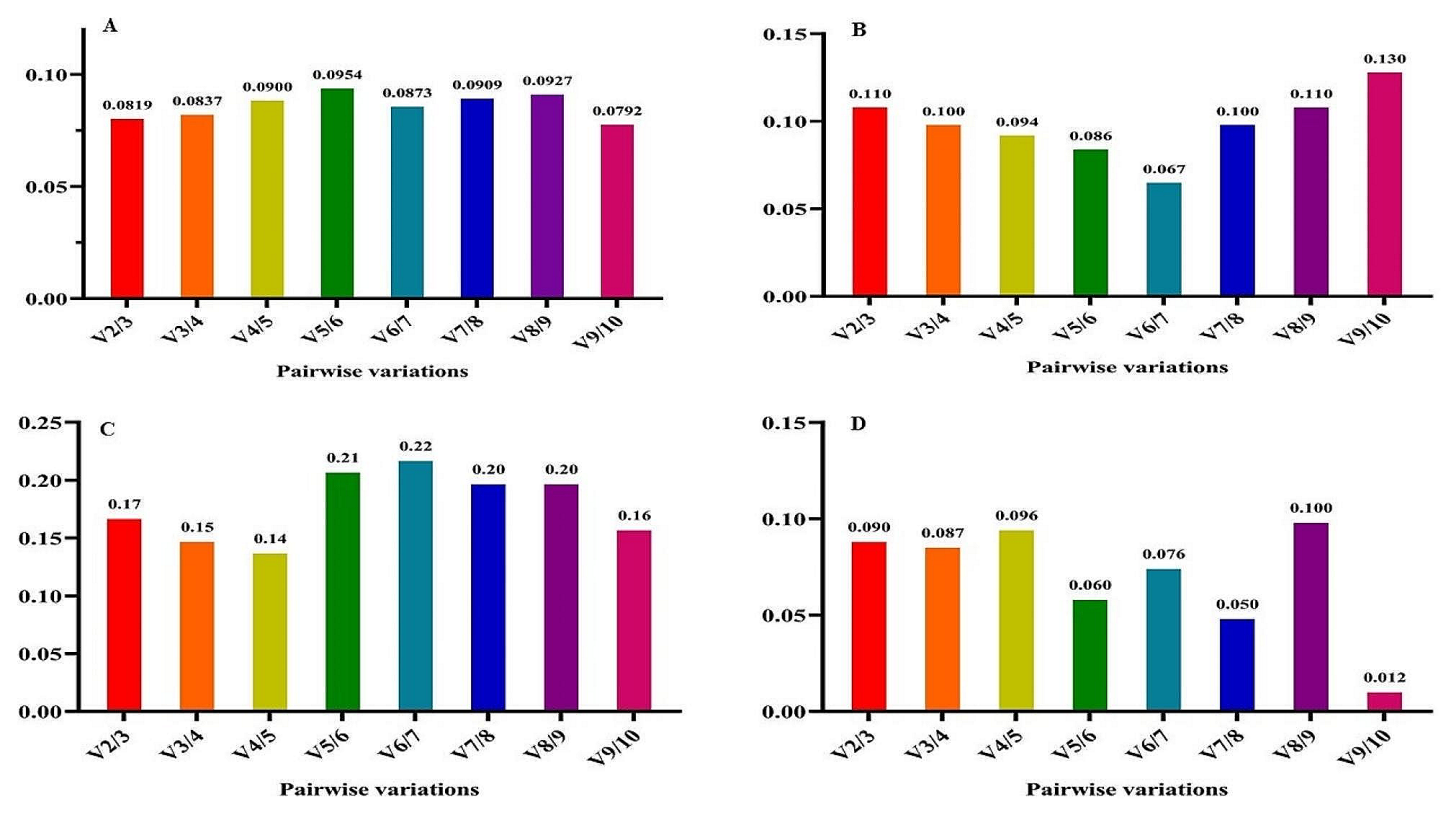
The pairwise variation values of ten reference genes under salinity stress obtained using GeNorm analysis. **A** TiO_2_ NPs + salinity stress, **B** TiO_2_ NPs + cold stress, **C** TiO_2_ NPs + high-temperature stress.

**Fig. 9:**
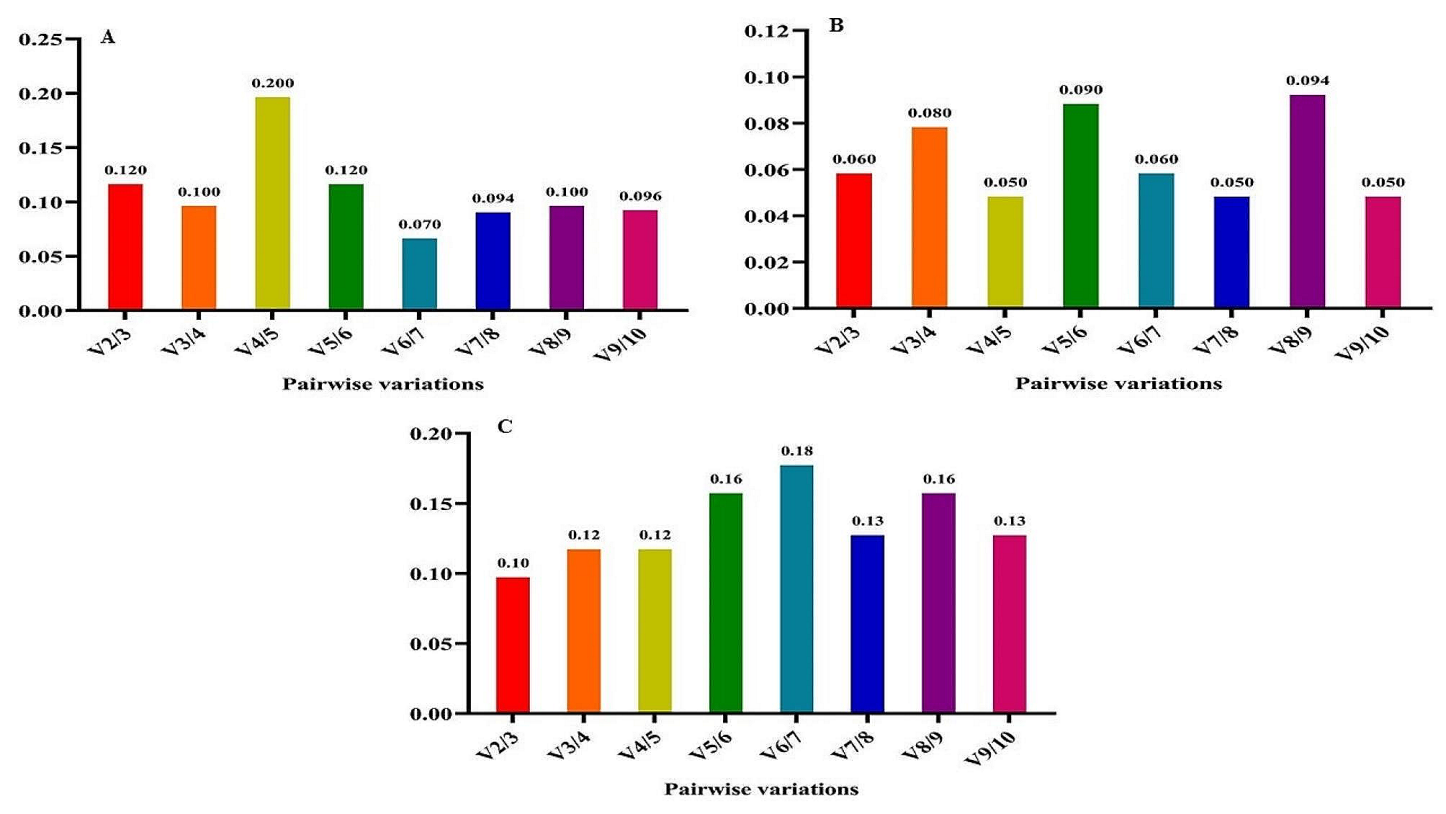
The pairwise variation values of ten reference genes under cold stress obtained using GeNorm analysis. **A** Cold plasma + salinity stress, **B** Cold plasma + cold stress, **C** Cold plasma + high-temperature stress.

**Fig. 10:**
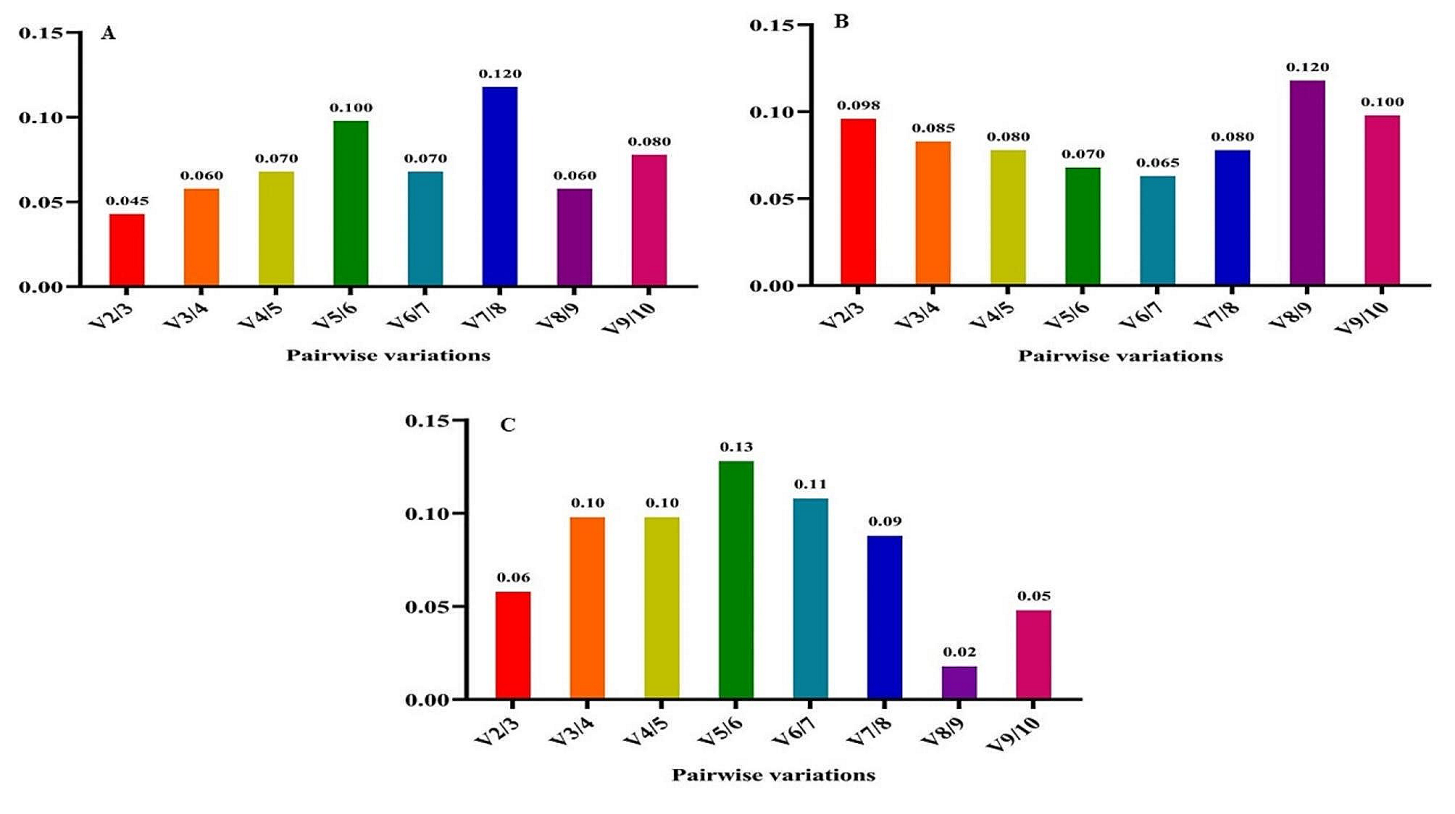
The pairwise variation values of ten reference genes under high-temperature stress obtained using GeNorm analysis. **A** EBR + salinity stress, **B** EBR + cold stress, **C** EBR + high-temperature

**Fig. 11:**
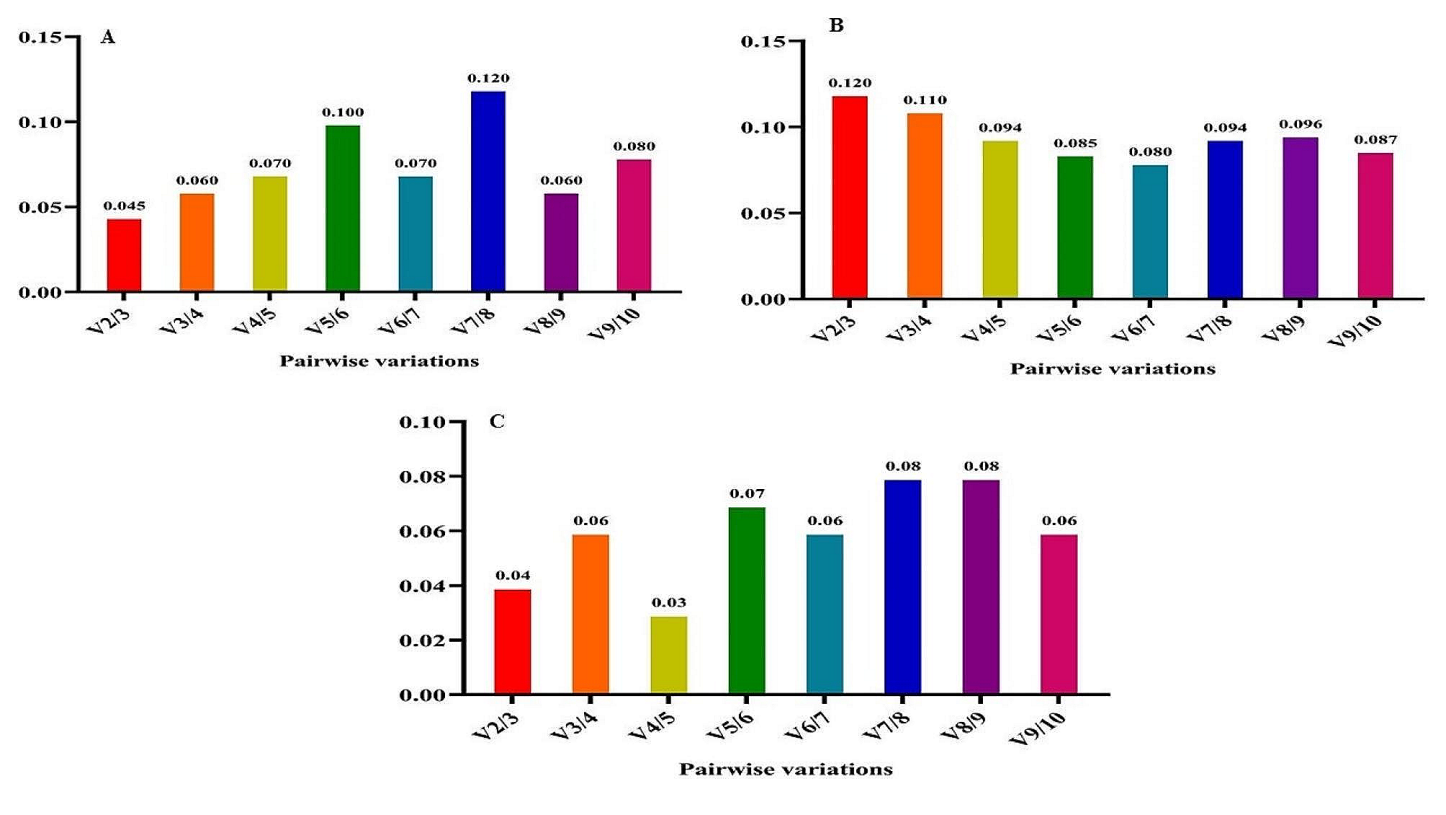
The pairwise variation values of ten reference genes under high-temperature stress obtained using GeNorm analysis. **A** Melatonin + salinity stress, **B** Melatonin + cold stress, **C** Melatonin + high-temperature

Nevertheless, when considering cold stress with TiO_2_ NPs treatment, all V to V5/6 values exceeded 0.15 (Fig. 8). Hence, it is crucial to validate the accuracy of the newly selected reference genes before commencing gene expression analysis in any research study. Validation under this treatment was effectively achieved with the first five genes (*β-tubulin*, *EEF-1α, UBE2D2, GAPDH*, and *Actin 11*). Furthermore, under cold plasma application with salinity stress and cold plasma application with high-temperature stress (Fig. 9), V4/5, and V5/6, V6/7 and V8/9 were above 0.15. Meanwhile, the V2/3 for the remaining experimental treatments was below 0.15 (Figs. 10 and 11). These results highlight the significance of employing suitable statistical methods, such as the GeNorm algorithm, to ascertain the optimal number of reference genes necessary for precise normalization in gene expression analysis.

### NormFinder analysis

The outcomes of the NormFinder analysis for the 10 candidate reference genes are presented in Table 4. The results highlight *EEF-1α* as the most stable gene, with a stability value of 0.580, surpassing the others. Additionally, the combination of *EEF-1α* and *β-tubulin*, with a stability value of 0.436, was suggested as a stable pair of genes applicable across all experimental treatments. In contrast, the *HSP70* gene, with a stability value of 1.8, was identified as the least efficient gene among the chosen ones. These results underscore the significance of employing suitable statistical tools, such as the NormFinder tool, to pinpoint the most stable reference genes for accurate gene expression analysis in fenugreek plants (Table 4). Analyzing the treatments individually yielded intriguing findings. In the control treatment, genes *EEF-1α, β-tubulin*, and *GAPDH* exhibited the lowest stability values, indicating their more stability. Conversely, genes *25 S rRNA, UbcH10*, and *HSP70* showed the highest stability values, designating them as the least stable genes. Similarly, in the treatment involving cold plasma application combined with cold stress, genes *EEF-1α, β-tubulin*, and *GAPDH* displayed the lowest stability values, suggesting their stability. On the other hand, genes *25 S rRNA, UbcH10*, and *Actin 11* demonstrated the highest stability values, suggesting their instability (Table 4).

### The ordering of reference genes determined by the outcomes from the three tools

For a comprehensive grasp of the effectiveness and stability of reference genes, Table 5 displays the ranking results derived from three tools utilized across the 13 experimental treatments in this study. Despite discrepancies in the results obtained from these tools, four genes *EEF-1α*, *β-tubulin*, *GAPDH*, and *eIF4A* emerged as stable genes, maintaining consistent rankings from 1 to 4 based on three tools. Conversely, genes *25 S rRNA*, *UbcH10*, *18 S rRNA*, and *Actin 11* were consistently identified as unstable genes, consistently holding rankings from 7 to 10 based on three tools. As an illustration, in the control treatment, genes *EEF-1α*, *β-tubulin*, *GAPDH*, and *eIF4A* were consistently classified among the four stable genes based on three tools. However, in the same treatment, the genes *25 S rRNA*, *UbcH10*, *18 S rRNA*, and *Actin 11* were simultaneously identified as the most unstable genes. Furthermore, in the treatment involving melatonin application along with cold stress, genes *EEF-1α*, *β-tubulin*, *GAPDH*, and *eIF4A* were classified among the top four stable genes based on three tools. Contrastingly, *25 S rRNA*, *UbcH10*, *18 S rRNA* and *Actin 11* genes were introduced as the most unstable genes (Table 5).

### Evaluation of target gene expression using stable and unstable reference genes

To validate the findings obtained from assessing the expression stability of reference genes, the expression profile of the *SSR* gene, a key gene involved in diosgenin biosynthesis, was analyzed. This section utilized the *EEF-1α*, *β-tubulin*, *EEF-1α* + *β-tubulin*, and *GAPDH* genes as the most stable genes, while the *UbcH10* and *25 S rRNA* genes employed as the least stable genes, serving as internal controls across all experimental treatments.

**Table 4:** Expression stability values of candidate reference genes calculated by NormFinder.

Treatment	Stability values									
	*EEF-1α*	*UBE2D2*	*β-tubulin*	*Actin 11*	*HSP70*	*GAPDH*	*UbcH10*	*eIF4A*	*25 S rRNA*	*18 S rRNA*
Control	0.433	1.369	0.817	2.300	2.555	0.859	2.613	1.700	2.694	1.607
Melatonin + Cold stress	0.512	1.991	0.352	0.959	1.439	0.938	1.229	1.560	1.856	1.406
Melatonin + High-temperature stress	0.565	1.120	0.825	2.116	2.083	0.817	1.017	0.950	2.070	2.451
Melatonin + Salinity stress	0.405	2.025	0.678	1.648	1.825	0.438	1.737	0.710	1.660	1.194
EBR + Cold stress	0.424	2.376	0.705	1.378	1.287	0.537	2.048	1.650	1.635	1.309
EBR + High-temperature stress	0.602	1.267	0.759	1.144	1.123	0.710	1.441	0.860	1.196	2.350
EBR + Salinity stress	0.593	1.618	0.416	1.328	1.076	1.015	2.331	0.540	1.552	1.183
TiO_2_ NPs + Cold stress	0.659	1.753	0.694	1.046	2.211	1.098	1.314	1.200	2.534	2.190
TiO_2_ NPs + High-temperature	0.445	1.007	0.578	2.595	1.673	0.933	1.384	1.250	1.874	1.804
TiO_2_ NPs + Salinity stress	0.546	1.154	0.488	1.513	1.357	0.644	1.890	0.990	2.391	1.232
Cold plasma + Cold stress	0.502	1.063	0.803	2.202	1.544	0.898	3.031	1.090	3.137	1.630
Cold plasma + High-temperature stress	0.715	1.097	0.840	1.871	2.352	0.793	3.000	1.450	2.980	2.800
Cold plasma + Salinity stress	0.606	2.206	0.625	1.082	2.609	0.672	2.783	0.450	3.183	1.370
Total samples	0.580	1.532	0.753	1.646	1.800	0.828	1.554	1.056	1.641	1.550

**Fig. 12:**
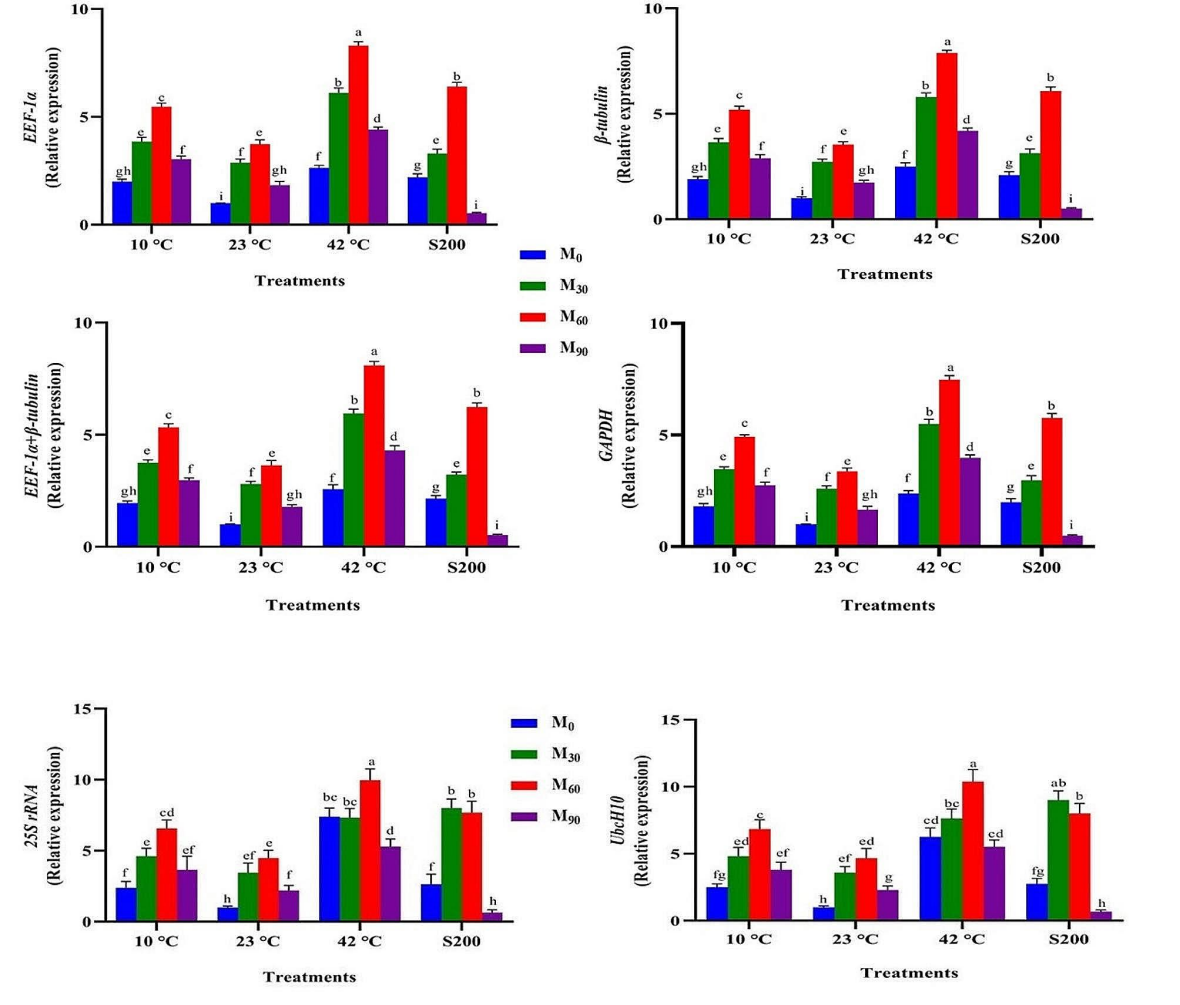
Illustrates the effects of various melatonin levels (M30, 60, and 90 ppm), temperature treatments (10, 23, and 42 °C), and salinity stress (200 mM) on the *SSR* expression. Duncan’s method was employed to compare the means at a 1% probability level, and columns with the same letters are not significantly different from each other

**Fig. 13:**
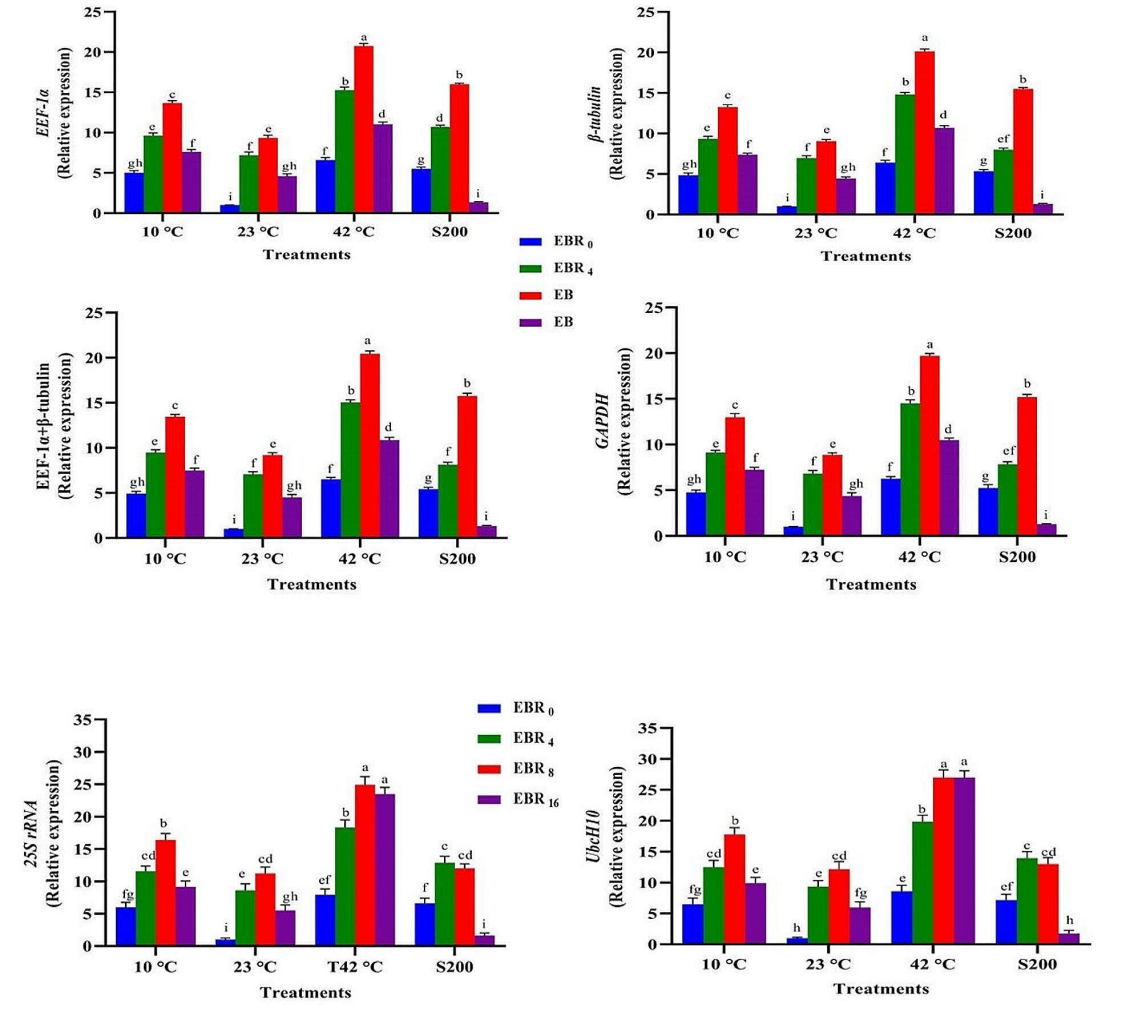
Illustrates the effects of various EBR levels (EBR0, 4, 8 and 16 µM), temperature treatments (10, 23, and 42 °C), and salinity stress (200 mM) on the *SSR* expression. Duncan’s method was employed to compare the means at a 1% probability level, and columns with the same letters are not significantly different from each other

**Fig. 14:**
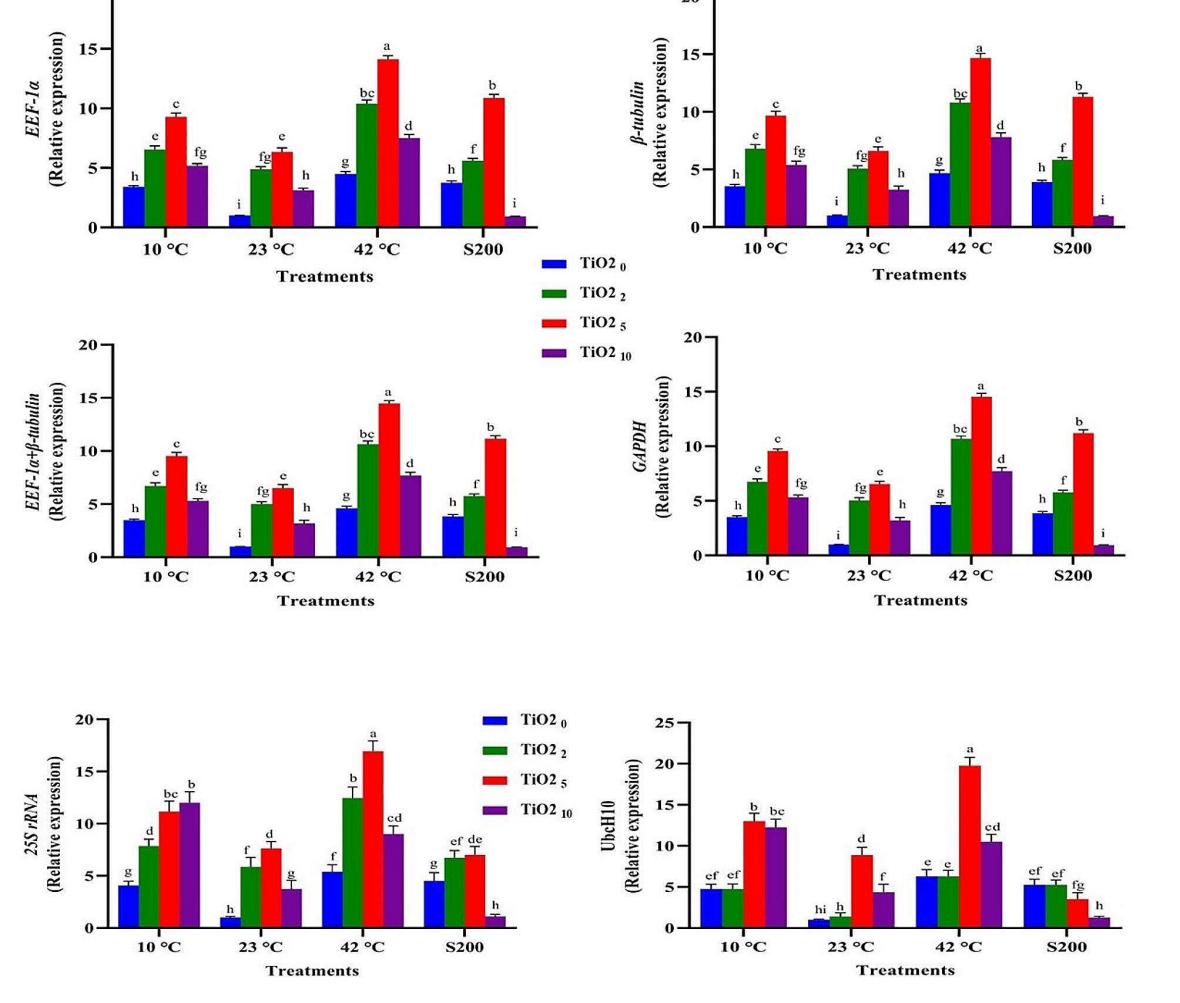
Illustrates the effects of various TiO_2_ NPs levels (0, 2, 5 and 10 ppm), temperature treatments (10, 23, and 42 °C), and salinity stress (200 mM) on the *SSR* expression. Duncan’s method was employed to compare the means at a 1% probability level, and columns with the same letters are not significantly different from each other

**Fig. 15:**
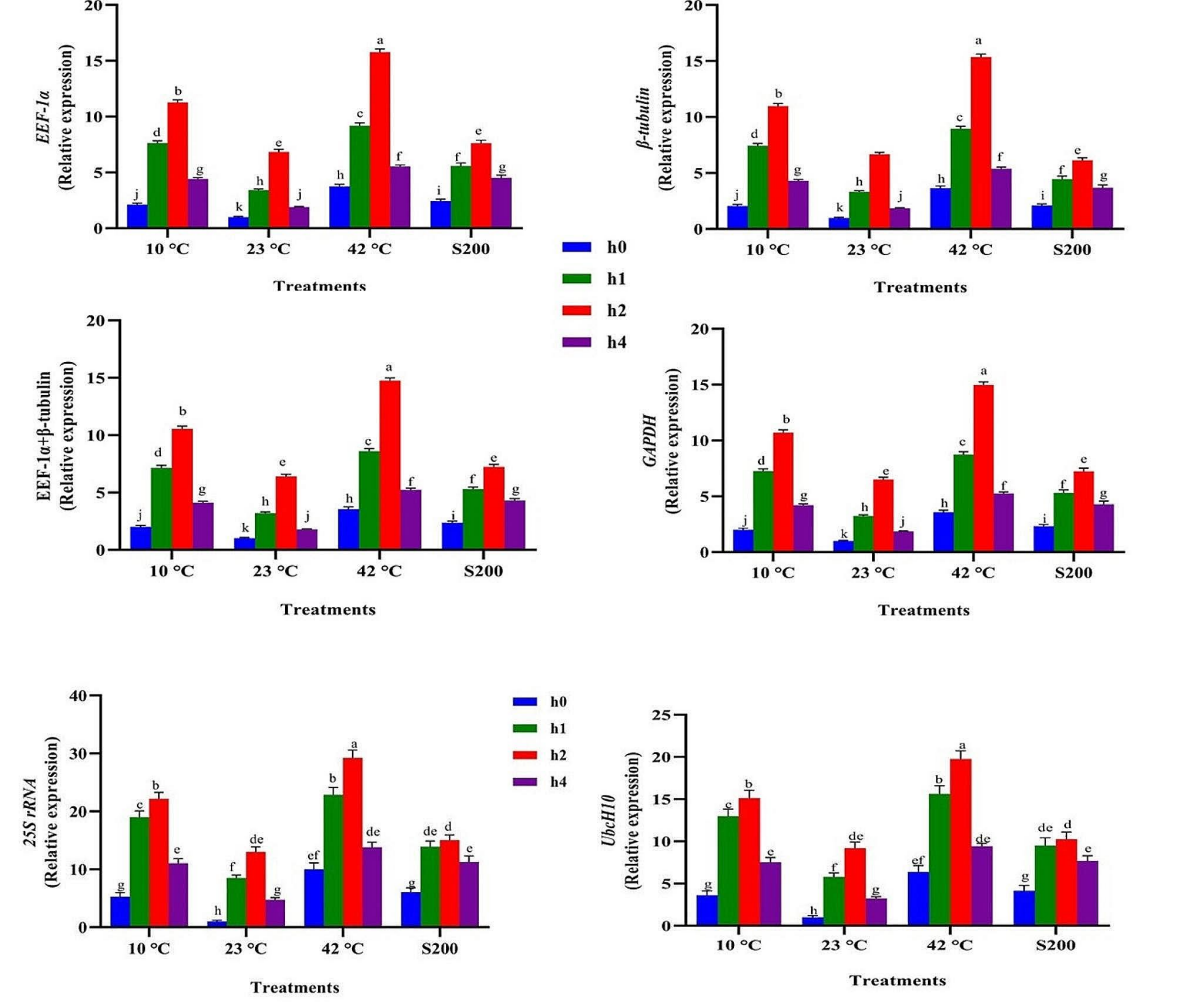
Illustrates the effects of exposure times to plasma (h 0, 1, 2, and 4 min), temperature treatments (10, 23, and 42 °C), and salinity stress (200 mM) on the *SSR* expression. Duncan’s method was employed to compare the means at a 1% probability level, and columns with the same letters are not significantly different from each other

The expression profiles of the *SSR* gene across 13 experimental treatments exhibited remarkable similarity when four stable reference genes (*EEF-1α*, *β-tubulin*, *EEF-1α* + *β-tubulin*, and *GAPDH*) were employed as internal controls (Figs. 12, 13 and 14, and 15). For instance, the highest and lowest levels of *SSR* gene expression were noted in plants treated with melatonin (60 ppm) under high-temperature stress and those grown under normal temperature conditions (Fig. 12). Similarly, the highest and lowest expression levels of this gene were observed in plants subjected to EBR treatment (8 µM) under high-temperature stress and those grown under normal temperature conditions, respectively (Fig. 13). In contrast, when unstable genes were used as internal controls (*UbcH10* and *25 S rRNA*) in the analysis of *SSR* gene expression under the 13 different treatments, a distinct pattern emerged. Specifically, the expression level of the *SSR* gene in plants treated with 8 µM EBR and subjected to salt stress (with stable genes as internal control) was twice as much as in plants treated with 4 µM EBR and exposed to salinity stress. On the contrary, utilizing unstable genes as the internal control revealed that the expression level of the *SSR* gene in plants treated with 8 µM EBR and exposed to salt stress was lower than in plants treated with 4 µM EBR under salt stress (Fig. 13).

To obtain a comprehensive understanding of the functionality of reference genes, an analysis of *SSR* gene expression was conducted across 148 different treatment combinations, employing both stable and unstable reference genes. The results illustrated that utilizing stable reference genes as internal controls maintained a consistent expression pattern of the *SSR* gene across identical experimental conditions. Conversely, employing unstable reference genes led to varying expression outcomes when compared to the results obtained with stable genes. For instance, when assessing the expression levels of *SSR* under high-temperature stresses (42 °C for 24 h) without melatonin, employing stable reference genes namely *EEF-1α*, *β-tubulin*, *EEF-1α* + *β-tubulin*, and *GAPDH* as internal controls resulted in corresponding expression values of 3.13, 3.04, 3.08, and 3.01, respectively. In contrast, the utilization unstable reference genes, namely *UbcH10* and *25 S rRNA*, led to expression values of 6.5 and 9, respectively (Supplementary Figs. 3, 4, 5, and 6). Furthermore, when investigating this gene expression levels under salt treatment (200 µM) with titanium dioxide (5 ppm), employing stable reference genes *EEF-1α, β-tubulin, EEF-1α + β-tubulin*, and *GAPDH* as internal controls resulted in expression values of 10.99, 11.25, 11.12, and 11.16, respectively. In contrast, the application of stable reference genes led to expression values of 3.5 and 6.2, respectively.

**Table 5:** Ranking order of the genes as determined by the outcomes from the three tools

Treatments	Rank *EEF-1α*			Rank *GAPDH*			Rank *β-tubulin*		
	BestKeeper	GeNorm	NormFinder	BestKeeper	GeNorm	NormFinder	BestKeeper	GeNorm	NormFinde
Control	1	1	1	2	2	3	3	3	2
Melatonin + Cold stress	1	1	2	3	4	3	2	3	1
Melatonin + High-temperature stress	1	1	1	3	4	2	2	3	3
Melatonin + Salinity stress	1	2	1	4	3	2	2	4	3
EBR + Cold stress	1	1	2	2	4	1	3	3	3
EBR + High-temperature stress	1	1	1	4	4	2	2	3	3
EBR + Salinity stress	1	1	3	2	2	4	4	6	1
TiO_2_ NPs + Cold stress	1	1	1	3	6	3	2	2	2
TiO_2_NPs + High-temperature	1	1	1	4	6	3	2	3	2
TiO_2_ NPs + Salinity stress	1	1	2	3	6	3	2	3	1
Cold plasma + Cold stress	1	3	2	2	1	1	3	2	4
Cold plasma + High-temperature stress	1	2	2	3	4	4	2	1	1
Cold plasma + Salinity stress	2	2	1	3	4	3	1	1	3

Treatments	BestKeeper	GeNorm	NormFinder	BestKeeper	GeNorm	NormFinder	BestKeeper	GeNorm	NormFinde
	Rank *eIF4A*			Rank *UBE2D2*			Rank *18 S rRNA*		
Control	4	4	8	5	5	10	6	7	6
Melatonin + Cold stress	4	2	8	5	6	10	9	9	6
Melatonin + High-temperature stress	4	2	4	5	8	6	8	9	10
Melatonin + Salinity stress	3	1	4	5	6	10	6	8	5
EBR + Cold stress	4	2	8	5	5	10	10	10	5
EBR + High-temperature stress	3	2	4	6	7	8	9	9	10
EBR + Salinity stress	3	3	2	8	9	9	7	10	6
TiO_2_ NPs + Cold stress	5	9	5	4	3	7	10	10	8
TiO_2_ NPs + High-temperature	5	10	5	3	2	4	8	9	8
TiO_2_ NPs + Salinity stress	7	10	4	3	2	5	10	9	6
Cold plasma + Cold stress	4	4	3	5	5	5	8	10	7
Cold plasma + High-temperature stress	5	9	5	4	3	3	10	10	8
Cold plasma + Salinity stress	4	3	2	5	5	7	8	10	6

Treatments	Rank *Actin 11*			Rank *HSP 70*			Rank *25 S rRNA*		
	BestKeeper	GeNorm	NormFinder	BestKeeper	GeNorm	NormFinder	BestKeeper	GeNorm	NormFinde
Control	7	8	4	8	6	7	9	10	9
Melatonin + Cold stress	6	5	4	7	10	7	8	7	9
Melatonin + High-temperature stress	6	5	9	7	6	8	10	7	7
Melatonin + Salinity stress	8	9	6	7	10	9	10	7	7
EBR + Cold stress	6	7	6	7	8	4	8	6	7
EBR + High-temperature stress	7	8	6	5	6	5	8	5	7
EBR + Salinity stress	7	8	7	5	5	5	9	7	8
TiO_2_ NPs + Cold stress	8	4	4	6	5	9	7	8	10
TiO_2_ NPs + High-temperature	6	4	10	7	5	7	10	8	9
TiO_2_ NPs + Salinity stress	5	4	8	6	5	7	9	8	10
Cold plasma + Cold stress	6	6	8	7	7	6	10	9	10
Cold plasma + High-temperature stress	7	5	6	6	6	7	8	8	9
Cold plasma + Salinity stress	7	6	5	6	7	8	9	9	10

Treatments	Rank *UbcH10*		
	BestKeeper	GeNorm	NormFinder
**Control**	10	9	9
Melatonin + Cold stress	10	8	5
Melatonin + High-temperature stress	9	10	9
Melatonin + Salinity stress	9	5	8
EBR + Cold stress	9	9	9
EBR + High-temperature stress	10	10	9
EBR + Salinity stress	6	4	10
TiO_2_ NPs + Cold stress	9	8	6
TiO_2_ NPs + High-temperature	9	7	6
TiO_2_NPs + Salinity stress	8	7	9
Cold plasma + Cold stress	9	8	9
Cold plasma + High-temperature stress	9	7	10
Cold plasma + Salinity stress	10	8	9

## Discussion

RT-qPCR is used as a wide method for gene expression analysis. To ensure the accuracy of gene expression studies, it is essential to design specific primers, select an appropriate fragment length, optimize PCR reaction conditions, and choose suitable internal controls [14]. The MIQE guidelines recommend using reference genes as the most suitable method for normalizing qRT-PCR data. However, the selection of appropriate reference genes for robust normalization of expression data in fenugreek is severely limited. Even though the expressions of reference genes are typically stable under normal conditions, they still play important roles in cellular processes. However, under certain conditions, such as in response to environmental stress or during different developmental stages, their expression levels may change. Therefore, it is crucial to carefully select appropriate internal reference genes based on their stability and expression levels under the specific experimental conditions being studied. This ensures that accurate and reliable gene expression analysis can be performed [21, 22]. In this study, we evaluated the suitability of candidate reference genes for gene expression analysis in fenugreek plants under different experimental conditions. To normalize qPCR results and obtain accurate Cq values of target genes, it is crucial to identify suitable internal control genes that exhibit minimal changes in expression levels across different experimental conditions and plant organs [23].

By employing descriptive statistics and measuring the mean Cq and SD, the stability of the housekeeping genes should be assessed initially. Based on our results, the *EEF-1α* gene demonstrated the highest level of expression among all samples, with a mean Cq value of 14.4 ± 0.68. On the other hand, the *25 S rRNA* gene showed the lowest expression level, with a mean Cq of 21.6 ± 2.86 (Fig. 1). Although measuring the mean Cq and SD may be tempting to rely on this method for evaluating gene stability due to its simplicity, it is only valid to a certain extent. In reality, the apparent simplicity of this method obscures biological and technical variations that must be taken into account [24]. Nicot et al. (2005) documented *EEF-1α* as the most stable gene in potato plants exposed to salt, cold, and late blight stress. Correspondingly, Saraiva et al. (2014) observed stable expression of *EEF-1α* under stress conditions and at various developmental stages of soybeans [25]. Guo et al. (2014) noted the consistent expression of *EEF-1α* in sugarcane plants under drought and salinity conditions [26] and documented the sustained expression of *EEF-1α* in pearl millet plants exposed to various abiotic environments [27]. The evaluation of the effectiveness of 10 reference genes across diverse experimental conditions in this study, covering 13 distinct treatments, using the BestKeeper tool, yielded results comparable to analyzing the efficiency of the same set of 10 reference genes across all experimental treatments (taking into account all treatments and treatment combinations). Despite slight differences and fluctuations, the *EEF-1α, β-tubulin, GAPDH*, and *eIF4A* genes were consistently identified as stable genes with minimal variation in both the simultaneous analysis and individual analysis of each treatment. Conversely, the *25 S rRNA, UbcH10, 18 S rRNA*, and *Actin 11* genes were consistently classified as unstable genes, demonstrating the greatest variability in both combined and individual analyses of each treatment (Table 2).

The optimal reference gene should display minimal variability in transcript levels across all experimental conditions. Numerous studies validating ideal reference genes in various crops have indicated significant variations in the expression of reference genes among different tissues, developmental stages, and experimental conditions in rice [28, 29], brassica [30], wheat [31], Arabidopsis [32, 33], maize [34], banana [35, 36], papaya [37], and apple [38, 39]. Furthermore, investigations on coffee and petunia plants in prior studies have illustrated significant variations in reference gene expression among different tissues within a specific genotype, as well as within the same tissue across different genotypes [40, 41]. These results affirm the importance of examining the stability of reference genes in all experimental samples to ensure precise gene expression analysis. Comparable alterations in the expression of reference genes in diverse organs, developmental stages, and experimental treatments have also been documented in other plant species. As an illustration, alterations in reference gene expression have been noted in potato plants subjected to drought, cold, and salt stresses [42], tomato plants exposed to nitrogen, cold, and light treatments [43], sunflower plants at six distinct stages of leaf development [44], wheat plants during interaction with the pathogen *Mycosphaerella graminicola* [45], and Populus plants at various stages of plant development [46]. These findings highlight the importance of selecting appropriate reference genes and evaluating their stability under diverse experimental conditions to ensure accurate and reliable gene expression analysis in plants. The findings of this study indicated that the expression patterns of three reference genes (*EEF-1α, β-tubulin, GAPDH)* were consistent across almost all treatments except excluding the TiO_2_ NPs application + cold stress, and cold plasma application + high-temperature and salinity treatments. Given the scarcity of established reference genes for gene expression investigations in fenugreek, researchers can leverage the outcomes of this study as a significant resource. It is crucial to note that this research was conducted specifically on fenugreek leaf samples, and therefore, caution should be exercised when extrapolating these results to studies involving other organs of the fenugreek plant, considering the distinct requirements for defining a reference gene in different conditions. Our research offers valuable insights into the choice of stable reference genes under various stress conditions in fenugreek plants. The identification of appropriate reference genes is crucial for precise gene expression analysis and the development of stress-tolerant fenugreek cultivars.

Identifying of stable reference genes and the determination of the optimal number necessary for precise normalization are crucial steps to enhance the accuracy and reliability of gene expression analysis in fenugreek plants. To ensure accurate comparison of gene expression levels, the application of multiple internal control genes, preferably at least two, has been recommended by Thellin et al. (1999) [47]. Nevertheless, Vandesompele et al. (2002) have presented persuasive reasons and evidence suggesting that the conventional normalization approach relying on a single internal control gene can yield inaccurate normalization outcomes [16]. These results underscore the significance of meticulously selecting internal control genes and employing suitable statistical methods to ascertain the optimal number of reference genes needed for precise normalization in gene expression analysis. The results of this study revealed that, although the three identified stable genes demonstrated satisfactory stability across all diverse experimental treatments, the expression of *EEF-1α, β-tubulin*, and *GAPDH* exhibited little variation in treatments involving TiO_2_ application with cold stress, cold plasma application with salinity, and high-temperature stresses (Table 4). The findings from the current study propose that a singular ideal reference gene applicable to all experimental conditions does not exist. Instead, choosing a suitable reference gene should be contingent upon the specific experimental conditions.

Our examination indicated variability in the expression stability of the candidate genes among the three statistical softwares used. Specifically, the BestKeeper software analysis identified *EEF-1α* and *GAPDH* as the most stable genes across all treatments. In contrast, the GeNorm and NormFinder tools revealed *β-tubulin* and *EEF-1α* as the most stable genes across the treatments. These results align with prior studies emphasizing the significance of choosing suitable reference genes tailored to specific experimental conditions. Given the fact that each statistical algorithm has unique analytical approaches, potentially conflicting results are reported from the same data at the same time. Furthermore, Contradictory results in previous studies in different species (*Cannabis Sativa, Actinidia deliciosa, Sorghum bicolor, Cichorium intybus, Vigna sinensis and Cannabis Sativa*) with different analytical programs are strong evidence for this claim [46, 48–52]. In this study, using three well-known statistical algorithms (BestKeeper, geNorm, NormFinder) ensures satisfactory results. In an investigation by Yang et al. (2015), for instance, *β-tubulin* served as a reference gene for normalizing qRT-PCR data related to *Salix matsudana* gene expression under salt and copper stresses [53]. In a study by Liu et al. (2014), *EEF-1α* genes were identified as the most stable reference genes across different growth stages and organs of ginseng [54]. Likewise, Wang et al. (2016) discovered that *EEF1-γ/IF3G1* genes exhibited the highest stability as reference genes across various tissues, whereas IF3G1/ACT11 emerged as the most stable reference genes in seedlings under heat stress conditions [55]. According to a report by de Jong et al. (2007), ribosomal protein genes consistently exhibit stable expression across a broad spectrum of experimental conditions, in contrast to conventional housekeeping genes like *GAPDH* [56]. Additionally, Sinha et al. (2015) documented that Tubulin (Tub) maintains stable expression levels during abiotic stress in pigeon pea [57]. Throughout numerous years, *GAPDH* has been regarded as a reliable and consistent reference gene across various organisms, frequently employed as an internal control in numerous expression studies [56, 58, 59]. Our study aligns with this perception, as we identified the *GAPDH* gene as one of the most stable housekeeping genes under all treatments. Consequently, *GAPDH* serves as a relatively stable reference gene.

Li and colleagues (2020) documented that the *β-tubulin* gene emerged as one of the most stable reference genes in licorice plants exposed to salinity and hormonal treatments [60]. Likewise, among the eight reference genes examined under various stress conditions in *Chrysanthemum morifolium*, *GAPDH* was identified as the most stable reference gene under heat stress, the fifth most stable gene under aphid infestation stress, and the third most stable gene under waterlogging stress. In a study by Cruz et al. (2009), *GAPDH* was selected as the most suitable reference gene for normalization in the leaves of different coffee cultivars out of the eight genes studied (Gu et al., 2011) [61, 62]. Furthermore, *GAPDH* was identified as a stable reference gene (Cq = 0.71) in pear plants under water deficit stress [3]. Collectively, our results underscore the importance of meticulous selection of appropriate reference genes and the utilization of suitable statistical methods to ensure accurate and reliable gene expression analysis in fenugreek plants across diverse experimental conditions.

*EEF-1α*, a GTP-binding protein, plays a crucial role in translation machinery and is widely considered a stable reference gene across different experimental conditions [63]. In an investigation on *Craterostigma plantagineum* subjected to water stress, Bartels and Juszczak (2017) recognized *EEF-1α* as the most stable reference gene in this plant [64]. In contrast, *18 S rRNA* was pinpointed as the least stable gene in their study. Our results align with other studies that have designated *18 S rRNA* as the most unstable reference gene in various organisms, including plants, fungi, and animals [63, 65–69]. These findings suggest that caution should be exercised while using *18 S rRNA* as a reference gene in gene expression analysis, and other suitable reference genes should be considered for accurate and reliable results. The research revealed fluctuations in gene expression stability among various genes in fenugreek under different experimental treatments, underscoring the absence of a universally applicable set of reference genes that exhibit stable expression under all environmental stress conditions. This finding is important because it highlights the need for careful selection of appropriate reference genes for accurate and reliable gene expression analysis in fenugreek (Tables 2 and 4, and 5).

Plants employ a range of strategies to cope with both biotic and abiotic stress, involving morphological, physiological, biochemical, and molecular responses. The specific patterns and intensities of these responses vary among different plant species and genera, influenced by factors such as the type and severity of stress and the inherent tolerance levels of the plants [70]. To develop plant varieties resilient to environmental stresses, a strategy involves investigating the response patterns of genes associated with plant resistance under various conditions. Among the cost-effective and efficient techniques available for this objective, real-time PCR stands out. To guarantee the precision of results derived from gene expression assessments, it is crucial to have access to internal controls, such as housekeeping genes. This is especially crucial because achieving consistent results in gene expression studies through real-time PCR can pose challenges for various reasons [10, 71, 72].

This study emphasizes the unique effects of each treatment on *SSR* gene expression, indicating significant variations in its behavioral patterns depending on specific elicitors. These varying responses underscore the crucial need for meticulous optimization of experimental conditions to ensure accurate results in gene expression studies utilizing qPCR. Indeed, selecting and utilizing stable reference genes across diverse experimental conditions pose a challenging task for researchers. The research demonstrated that the expression pattern of this gene remained completely uniform across all 13 experimental treatments and their combinations when stable genes were utilized. In contrast, using unstable reference genes led to fluctuations in the gene’s expression pattern, evident in both the 13 treatments and the 148 treatment combinations, unlike when stable genes were utilized (Figs. 12, 13 and 14, and 15). The findings of this study indicate that employing only two or three reference genes is sufficient for approximately 148 different treatments. Therefore, it is emphasized that employing an improper and unsuitable reference gene could lead to misinterpretations in entire gene expression profiling experiments, ultimately producing unreliable outcomes. Ebrahimibasabi et al., (2020), Arabasadi et al., (2024), Sheikhi et al. (2023) and Mohamadi Esboei et al. (2022) demonstrated that the application of cold plasma, cold plasma along with melatonin, EBR and melatonin combined with exposure to high-temperature and salinity stresses, led to increased *SSR* gene expression and notably enhanced diosgenin content in fenugreek [4, 6, 7, 13]. In these studies, the application of cold plasma, cold plasma with melatonin, EBR along with high-temperature stress, and melatonin in combination with salt stress resulted in *SSR* gene expression increases of 5.5, 21, 6.5, and 11 times, respectively, compared to the control plants [4, 6, 7, 13]. As the pioneering study in identifying stable reference genes in fenugreek, this research can serve as a valuable model for future assessments conducted by researchers exploring gene expression in fenugreek.

## Conclusion

To our knowledge, despite the abundance of transcriptome data for fenugreek, no comprehensive analysis of large-scale gene expression datasets has been undertaken to pinpoint stable reference genes. This study aims to identify promising housekeeping gene candidates that could serve as reliable reference genes for quantitative gene expression analysis. To obtain a comprehensive understanding of the efficiency and stability of reference genes, the data from this study were evaluated on an individual basis for each of the 13 treatments and collectively for 148 unique treatment combinations using three tools. We ranked well-known reference genes based on their stability, and our results indicated that a combination of the *EEF-1α*, *β-tubulin*, and *GAPDH* genes is suitable for normalizing gene expression data in fenugreek. Additionally, we found that the *UbcH10*, *25 S rRNA*, and *18 S rRNA* and genes were the most unstable among the candidate genes. While the outcomes from all three tools were remarkably consistent in identifying both stable and unstable reference genes in both individual treatment analysis and the analysis of all samples, there were slight variations in the results. It’s crucial to highlight that the stable reference genes identified by the three tools showed some fluctuations in specific treatments, such as cold stress with TiO_2_ NPs application, cold plasma application with salinity stress, and cold plasma application with high-temperature stress treatments, compared to others. Therefore, we recommend utilizing at least two reference genes with high stability to normalize gene expression in fenugreek. Furthermore, we suggest that the ranking of reference genes in this study should be used to determine the stability of reference genes in other tissues and under different conditions for future studies. This systematic analysis of reference genes offers valuable insights for researchers conducting gene expression studies, especially under different abiotic stress conditions and elicitor treatments. It contributes to facilitating more robust and standardized analyses not only in fenugreek but also in other plant species.

### Electronic supplementary material

Below is the link to the electronic supplementary material.

Supplementary Material 1

## Data Availability

No datasets were generated or analysed during the current study.
