# Supplementary material for: Characterizing reference genes for high-fidelity gene expression analysis under different abiotic stresses and elicitor treatments in fenugreek leaves
Source: Plant Methods. 2024 Mar 16;20:40. doi: 10.1186/s13007-024-01167-6 (PMC10943880; doi:10.1186/s13007-024-01167-6)
Supplement: Supplementary file 1 — Supplementary Material 1 [file 13007_2024_1167_MOESM1_ESM.docx]

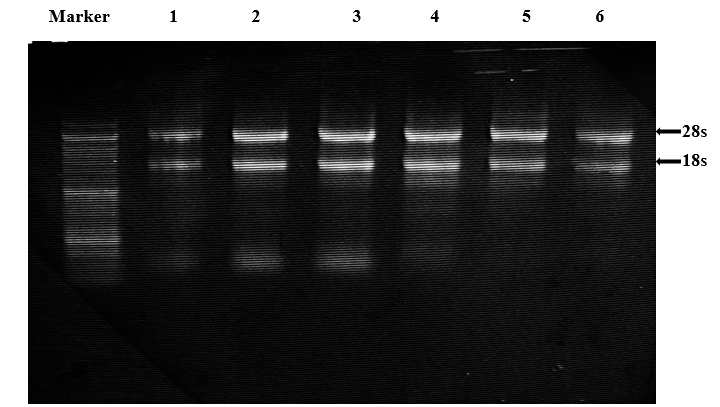


**Figure S1.** RNA gel electrophoresis of sample sets.


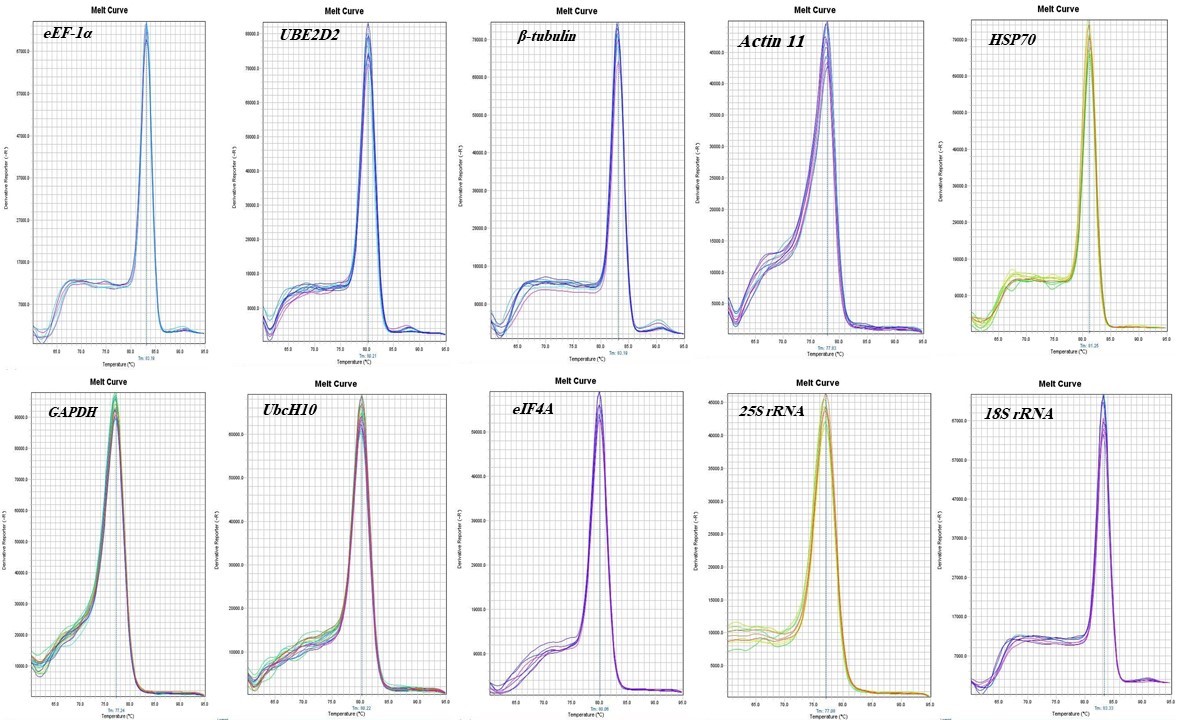


**Figure S2.** Melt curves of the ten candidate reference genes. Note: X-axis: Temperature (℃); Y -axis: Derivative Reporter (-Rn).


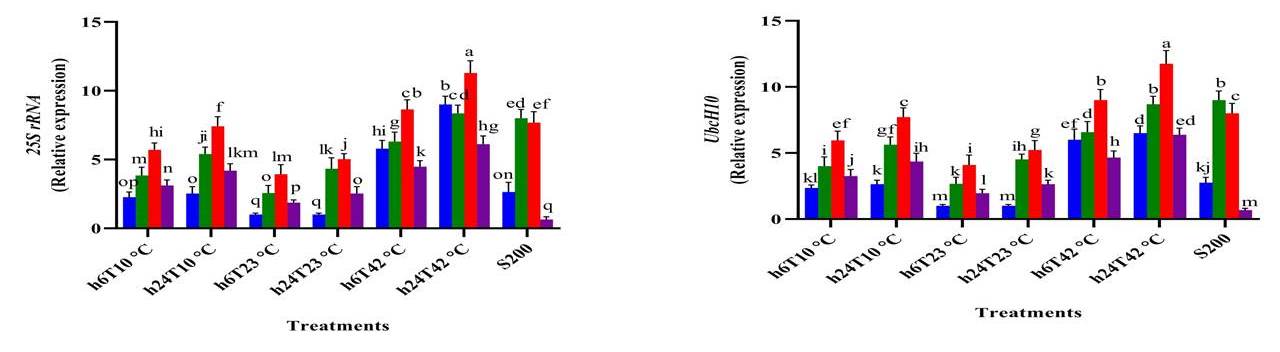

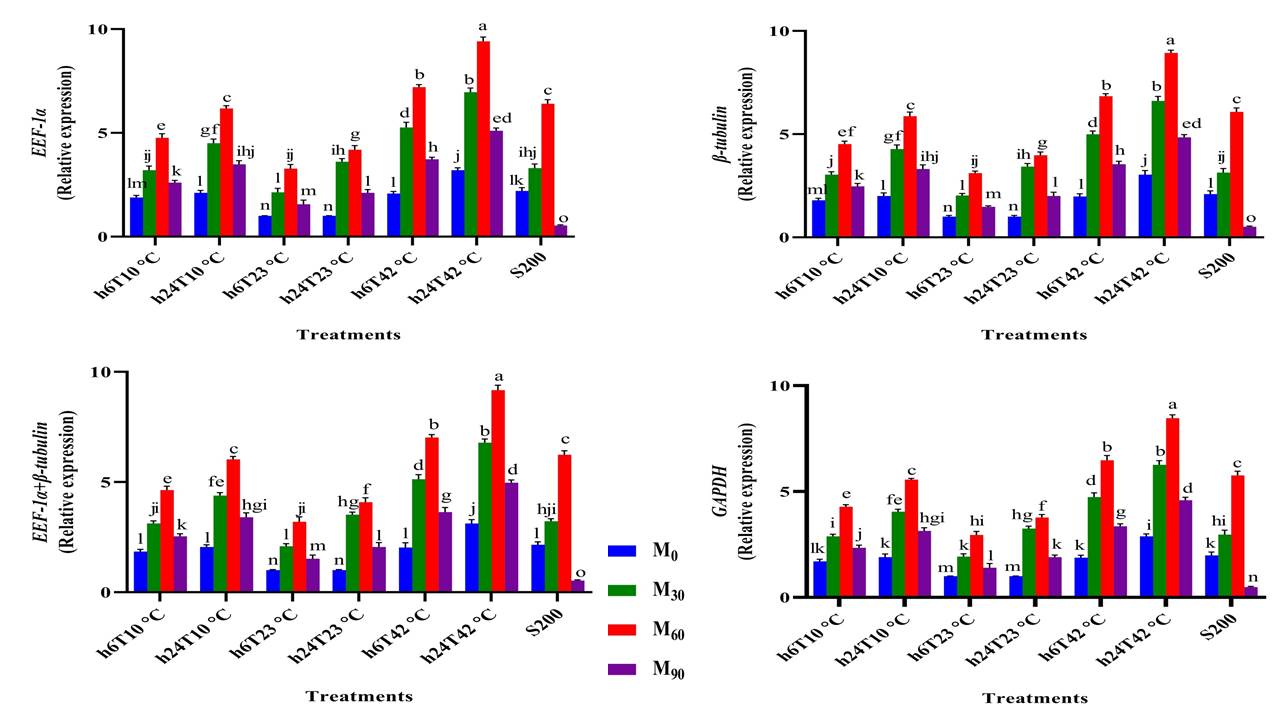


**Figure S3.** Illustrates the effects of various melatonin levels (M30, 60, and 90 ppm), temperature treatments (10, 23, and 42 ^°^C), time courses (6 and 24 h), and salinity stress (200 mM) on the *SSR* expression. Duncan's method was employed to compare the means at a one percent probability level, and columns with the same letters are not significantly different from each other.

**
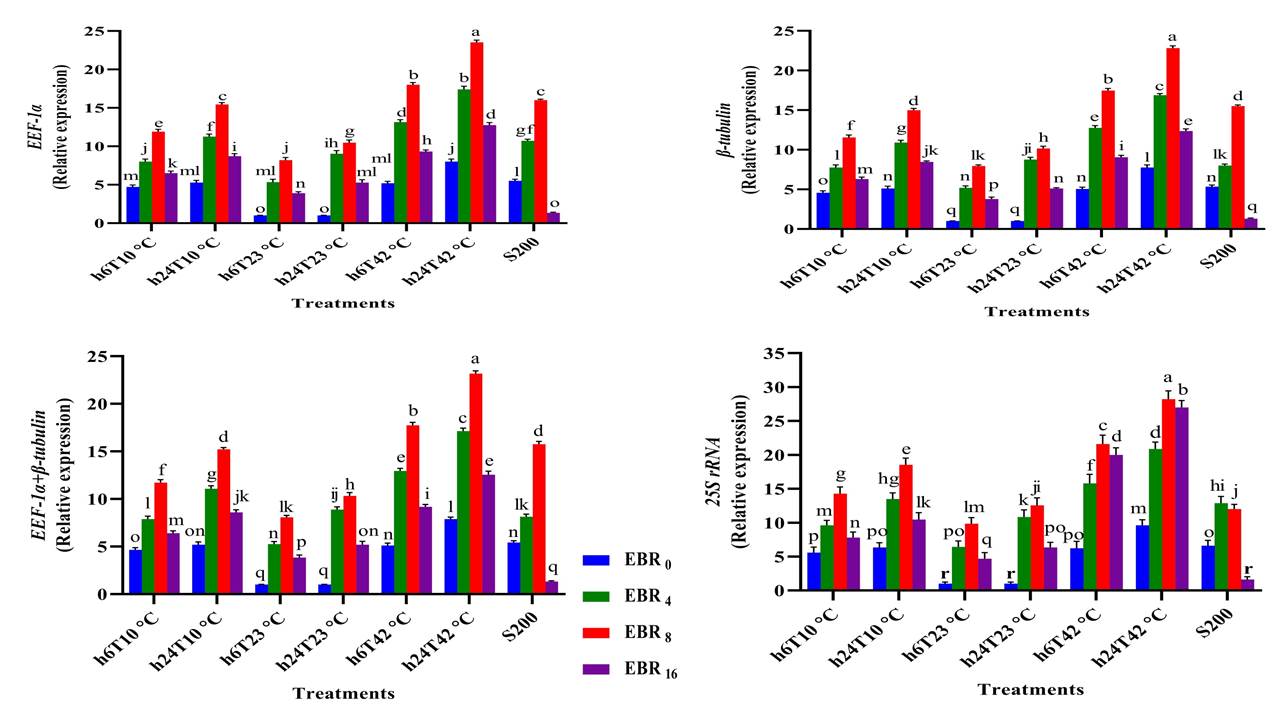
**

**
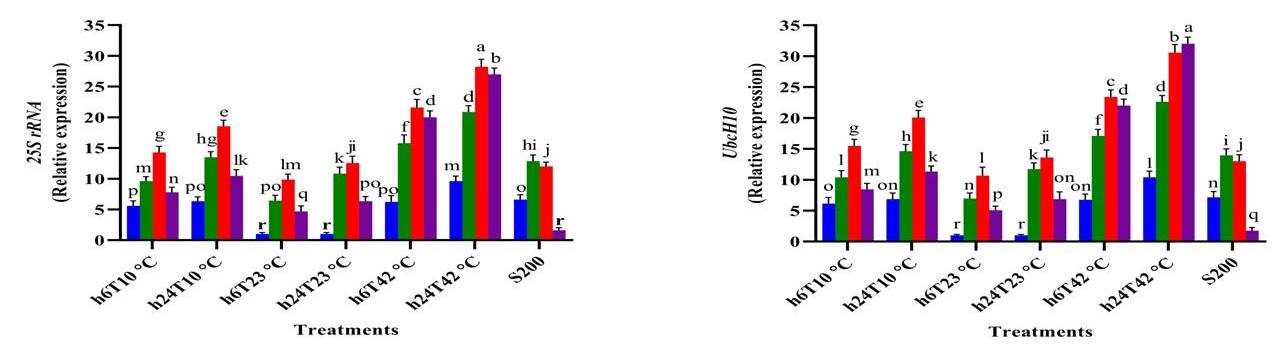
**

**Figure S4.** Illustrates the effects of various EBR levels (EBR 0, 4, 8 and 16 µM), temperature treatments (10, 23, and 42 ^°^C), time courses (6 and 24 h), and salinity stress (200 mM) on the *SSR* expression. Duncan's method was employed to compare the means at a one percent probability level, and columns with the same letters are not significantly different from each other.


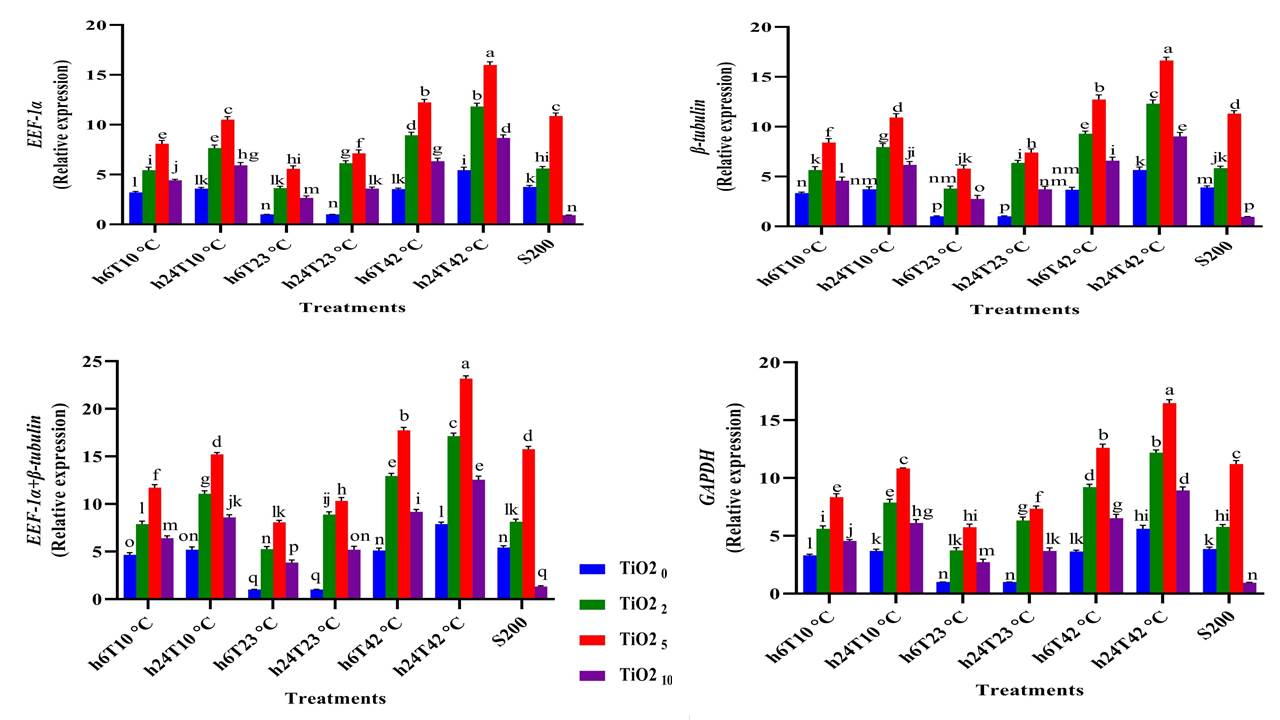


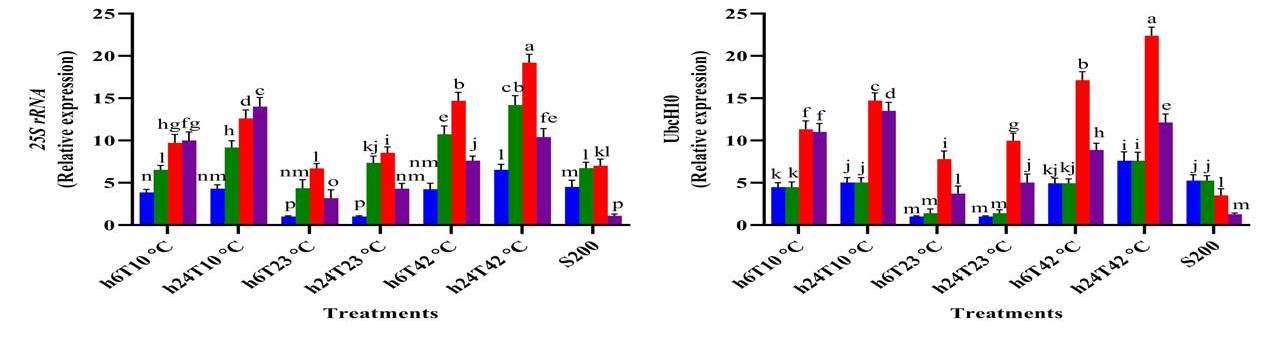


**Figure S5.** Illustrates the effects of various TiO_2_ NPs levels (0, 2, 5 and 10 ppm), temperature treatments (10, 23, and 42 ^°^C), time courses (6 and 24 h), and salinity stress (200 mM) on the *SSR* expression. Duncan's method was employed to compare the means at a one percent probability level, and columns with the same letters are not significantly different from each other.


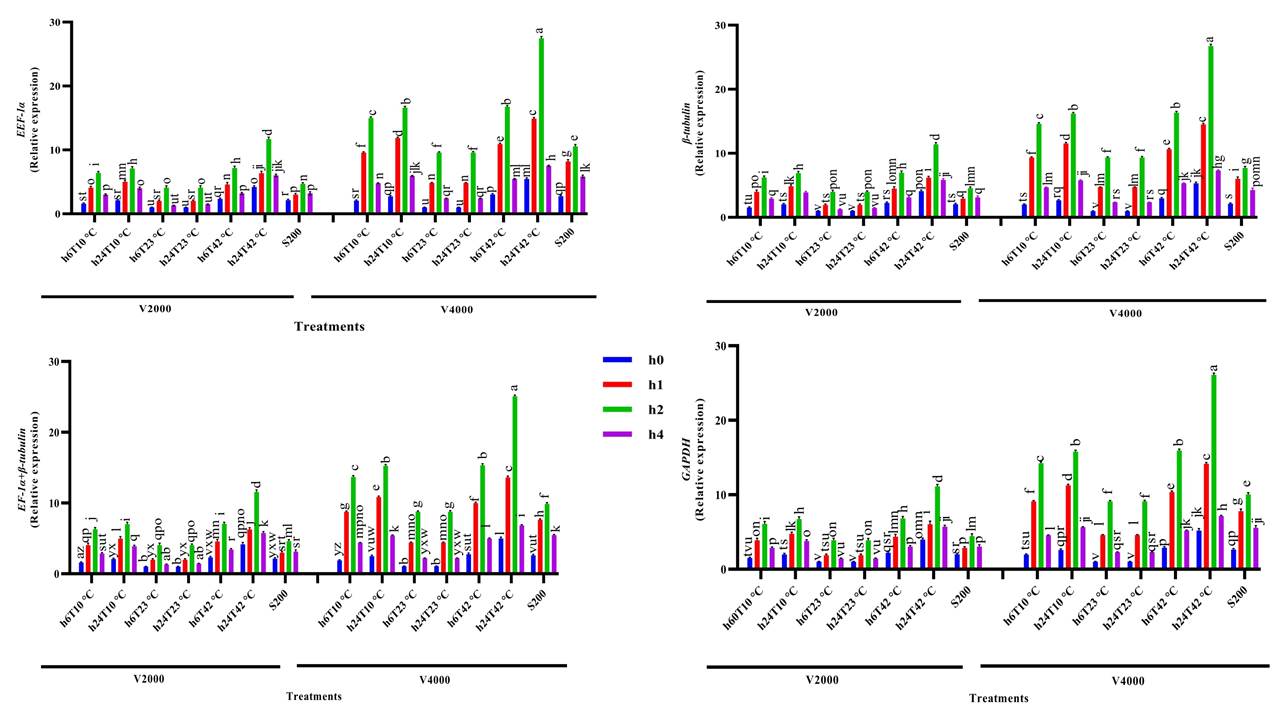


**Figure S6.** Illustrates the effects of various cold plasma levels (2000 and 4000 V), exposure times to plasma (h 0, 1, 2, and 4 min), temperature treatments (10, 23, and 42 ^°^C), time courses (6 and 24 h), and salinity stress (200 mM) on the *SSR* expression. Duncan's method was employed to compare the means at a one percent probability level, and columns with the same letters are not significantly different from each other.


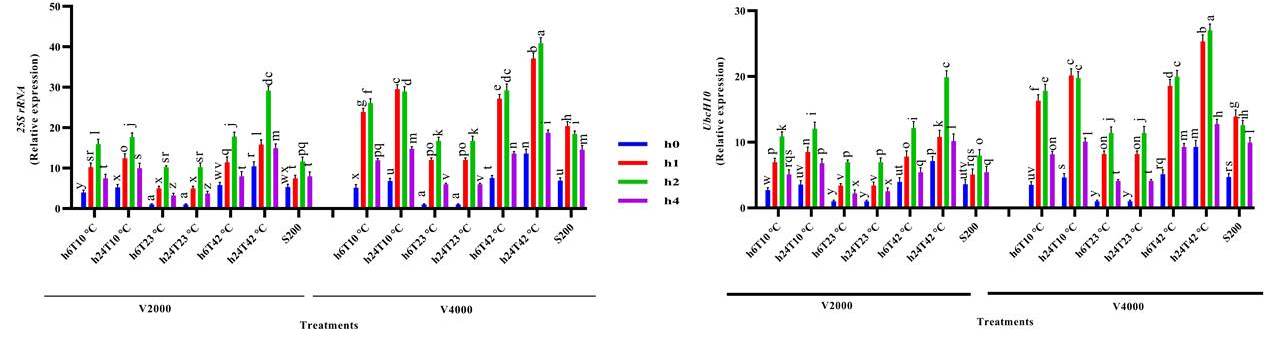


**Figure S6.** Illustrates the effects of various cold plasma levels (2000 and 4000 V), exposure times to plasma (h 0, 1, 2, and 4 min), temperature treatments (10, 23, and 42 ^°^C), time courses (6 and 24 h), and salinity stress (200 mM) on the *SSR* expression. Duncan's method was employed to compare the means at a one percent probability level, and columns with the same letters are not significantly different from each other
